# Generative Forecasting of Longitudinal 3D-OCT Under Anti-VEGF Therapy for Diabetic Macular Edema

**DOI:** 10.3390/diagnostics16203382

**Published:** 2026-10-17

**Authors:** Yuxiang Feng, Chaofeng Liang, Shujun Wang

**Affiliations:** 1 College of Control Science and Engineering, Zhejiang University, Hangzhou 310027, China; fengyx@zju.edu.cn; 2 Department of Neurosurgery, The Third Affiliated Hospital of Sun Yat-Sen University, Guangzhou 510630, China; lcfjeff@163.com; 3 Department of Biomedical Engineering and Sports Technology, The Hong Kong Polytechnic University, Hong Kong, China

**Keywords:** diabetic macular edema, diabetic retinopathy, optical coherence tomography, generative forecasting, anti-VEGF therapy, discrete latent dynamics

## Abstract

**Background/Objectives:** Forecasting the individual anatomical response to anti-vascular endothelial growth factor (anti-VEGF) therapy from optical coherence tomography (OCT) could support the management of diabetic retinal disease. Direct volumetric generation is difficult because treatment-associated change is local while most of the retina is stationary, so generative models regress to the mean and blur retinal layers. **Methods:** We propose a two-stage framework that decouples baseline anatomy from longitudinal change in 3D-OCT. A Retinal Response Tokenizer compresses the latent residual between baseline and follow-up volumes into discrete action tokens via Finite Scalar Quantization (FSQ), and a conditional decoder renders the follow-up volume from baseline features and these tokens. An Action Transformer predicts the tokens from the baseline scan, the follow-up interval, and baseline covariates. **Results:** On the OLIVES trial cohort (diabetic retinopathy and macular edema; 738 test pairs from five held-out eyes), our method achieved the highest structural similarity in every test eye (SSIM 0.657 vs. 0.543–0.623) and tracked the central subfield thickness (CST) change measured by a frozen regressor (Δ-r=0.88). In a five-fold cross-validation over 52 eyes, it improved PSNR over an identity copy by 1.27 dB (95% CI 0.93–1.57) and reduced the CST change error by 10.5 μm (95% CI 3.1–19.4). **Conclusions:** Prospective validation is required before clinical use.

## 1. Introduction

Diabetic macular edema (DME) is the leading cause of moderate-to-severe visual impairment among working-age adults worldwide, resulting from blood–retinal barrier breakdown and microvascular leakage [1,2]. Intravitreal anti-vascular endothelial growth factor (anti-VEGF) agents, such as ranibizumab, aflibercept, and bevacizumab, serve as the standard first-line pharmacotherapy [3,4], and they are also used to improve diabetic retinopathy (DR) severity in eyes without center-involved DME [5]. Repeated injections aim to resolve intraretinal and subretinal exudative fluid, restore retinal layer organization, and prevent irreversible photoreceptor loss. Nevertheless, treatment responses vary substantially in both clinical practice and multi-center randomized trials of anti-VEGF agents in DME and neovascular age-related macular degeneration [6,7]. Persistent macular thickening remains common, affecting approximately one-third to two-thirds of eyes after 24 weeks of anti-VEGF therapy depending on the agent [8], and many eyes show a suboptimal early anatomical response [9]; these patients are exposed to treatment fatigue, cumulative financial burden, and procedural risks including endophthalmitis and intraocular pressure spikes. Because structural features, such as disorganization of the retinal inner layers (DRIL) and localized intraretinal cystoid fluid volume, correlate with functional visual prognosis [10,11], forecasting the patient-specific 3D anatomical change could eventually support individualized monitoring; whether it can inform treatment decisions has to be established prospectively.

Longitudinal 3D retinal forecasting presents several technical challenges. Deep learning has proven effective for diagnostic screening and referral triaging from cross-sectional OCT scans [12,13]. Subsequent community benchmarks, notably the RETOUCH challenge [14], catalyzed automated detection and volumetric quantification of fluid compartments across scanner platforms [15]. Predictive studies have also modeled disease recurrence and treatment requirements [16,17,18]. However, existing prognostic approaches have predominantly relied on discriminative models to predict scalar clinical endpoints, such as future injection frequency, categorical responder status, or tabular visual acuity scores [19,20,21,22,23,24]. While clinically informative, scalar predictions lack anatomical detail: they cannot show where intraretinal cystoid fluid pockets will resolve, whether the foveal depression will re-establish, or how the ellipsoid zone (EZ) and retinal pigment epithelium (RPE) layers will reorganize under pharmacotherapy.

Deep learning is increasingly applied across ophthalmic imaging [25], and generative models offer a path toward image-based forecasting by synthesizing post-treatment scans directly. Generative adversarial networks (GANs) were initially introduced for unsupervised anomaly detection and normative representation learning in retinal OCT (e.g., f-AnoGAN) [26], followed by synthetic B-scan generation for data augmentation [27,28] and medical education [29] and cross-modality synthesis between structural OCT and OCT angiography [30,31]. More recently, advanced diffusion models have been developed for content-preserving anterior-segment OCT despeckling [32] and lesion-conditioned synthesis (such as RetiDiff) [33], alongside latent diffusion models (LDMs) [34] and cascaded architectures exploring high-resolution volumetric synthesis [35]. In ocular pharmacotherapy, conditional image translation frameworks, including pix2pix [36] and CycleGAN [37], have been explored to forecast post-injection retinal cross-sections [38]. However, existing generative frameworks suffer from key limitations in interventional settings: they either generate cross-sectional 2D slices independently without inter-slice 3D continuity [28,38], or are not conditioned on the same patient’s baseline scan [26,33]. When established generative frameworks originally developed for 2D image synthesis (including CycleGAN [37], conditional diffusion Palette [39], and Diffusion Transformers I2I-DiT [40]) are adapted for direct longitudinal 3D-OCT forecasting, alongside spatiotemporal progression architectures [41], they encounter a clear empirical limitation. Under treatment, fluid resolution changes the retina locally, whereas most of the volume is anatomically stationary: in the OLIVES cohort, a median of 94% of retinal tissue voxels change by less than the inter-visit noise floor (Section 3.6). When conventional continuous models are tasked with monolithic, end-to-end 3D synthesis, the optimization objective is dominated by the stationary background. As demonstrated in our experiments, these models regress to the mean, generating smoothed retinal features and blurring subtle laminar microstructures. Direct volumetric baselines yield structural similarity scores (SSIM 0.547–0.567) that fall below a static identity copy (SSIM 0.604). More importantly, their synthesized scans can decouple from the downstream biomarkers: CycleGAN achieves perceptual sharpness but produces a CST change correlation of merely Δ-r=0.465 and a negative correlation for the OCT-derived BCVA change (Δ-r=−0.166). These empirical findings indicate that predicting the monolithic future volume directly forces the network to allocate capacity to stationary background, obscuring localized change.

**Figure 1:**
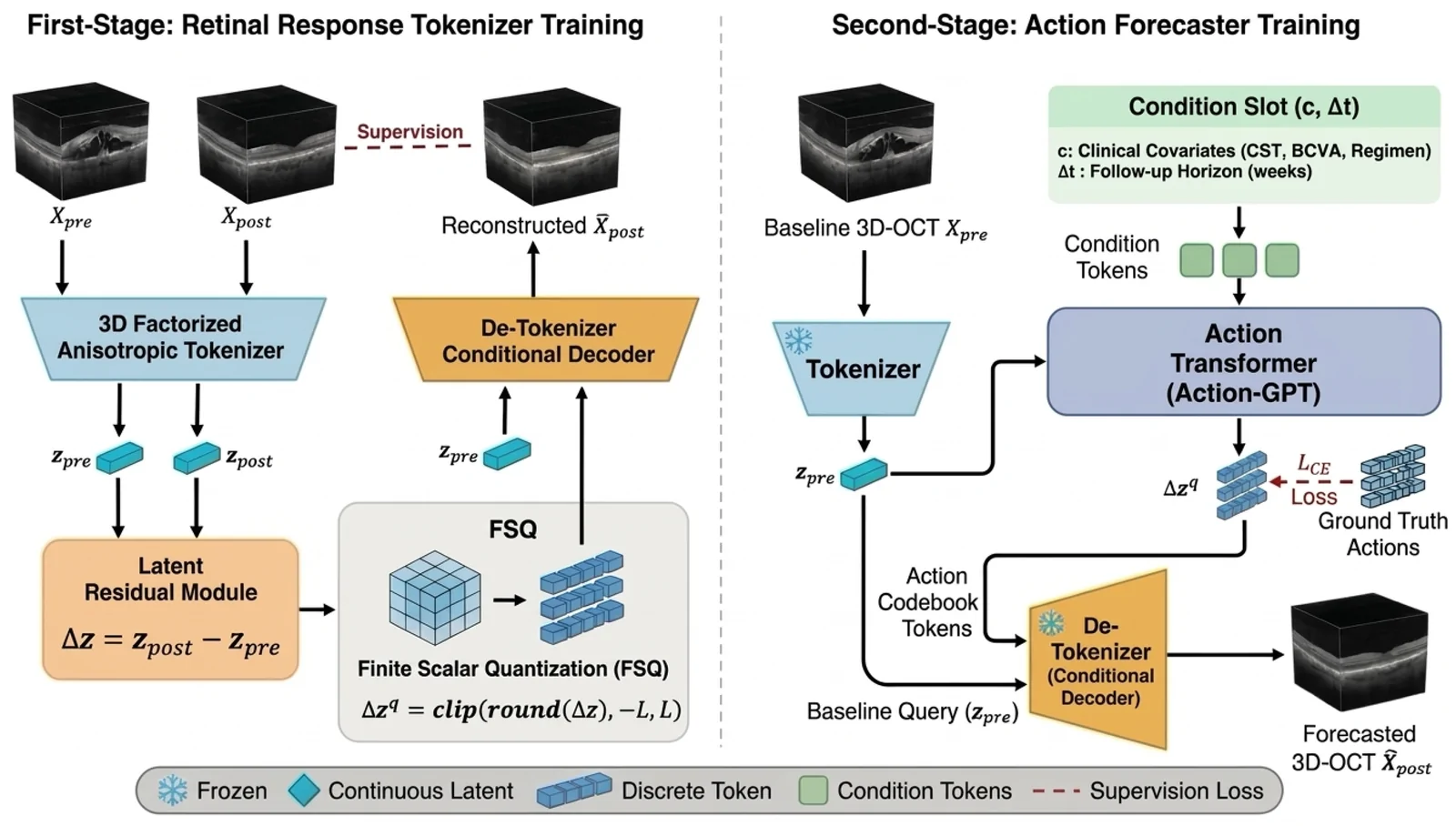
Schematic architecture of the proposed decoupled generative forecasting framework. (**Left**) Stage 1, Retinal Response Tokenizer: Paired longitudinal 3D-OCT scans $X_{\mathrm{pre}}$ and $X_{\mathrm{post}}$ are encoded by a factorized 3D tokenizer E into continuous latents $z_{\mathrm{pre}}$ and $z_{\mathrm{post}}$ (cyan slabs; cyan diamond in the legend). Their latent residual is quantized by Finite Scalar Quantization (FSQ) into discrete action tokens $\Delta z^{q}$ (segmented blue blocks), and the De-Tokenizer D reconstructs ${\hat{X}}_{\mathrm{post}}$ with $z_{\mathrm{pre}}$ as cross-attention queries and $\Delta z^{q}$ as keys/values. (**Right**) Stage 2, Prognostic Action Generator: the frozen tokenizer (snowflake icon) encodes the baseline scan, and an Action Transformer predicts $\Delta z^{q}$ in a single forward pass, conditioned on the baseline covariates *c* (CST, BCVA, regimen) and the follow-up interval Δt (condition tokens, green boxes); the frozen De-Tokenizer then synthesizes the forecast ${\hat{X}}_{\mathrm{post}}$. Red dashed lines denote the training losses ($L_{\mathrm{Stage}1}$ and $L_{\mathrm{CE}}$). Details are given in Section 2.2 and Section 2.3 and Supplementary Figure S1.

To address this limitation, we argue that prognostic generation benefits from explicit decoupling: the stationary baseline anatomical skeleton must be anchored, while representational capacity is concentrated on treatment-associated, localized change. We propose a two-stage generative forecasting framework designed specifically for longitudinal 3D-OCT under anti-VEGF therapy (Figure 1). In Stage 1, a Retinal Response Tokenizer uses a factorized spatio-depth 3D transformer to address the anisotropic resolution of clinical OCT volumes, adapting factorized spatio-temporal attention [42] to depth-raster OCT geometry. The tokenizer explicitly extracts the latent longitudinal residual between follow-up and baseline. We compress this residual into a sequence of compact, discrete action tokens via Finite Scalar Quantization (FSQ) [43], which provides a fixed, collapse-free discrete vocabulary at negligible fidelity cost. A conditional 3D decoder then queries the baseline anatomy to reconstruct the post-treatment volume. In Stage 2, a prognostic action generator operates solely on pre-treatment baseline OCT volumes, baseline clinical covariates, and scheduled follow-up intervals (Δt), formulating forecasting as discrete latent action prediction over localized trajectories [44]. To keep the learned vocabulary small for a cohort of a few dozen eyes, the generator predicts only the coarse part of each FSQ token, anchored by an empirical positional prior, and draws the fine part from training statistics.

Evaluating synthetic medical volumes solely through perceptual image metrics is insufficient, and a cohort with few independent eyes requires eye-level statistics. We therefore benchmark all models across three complementary dimensions: (i) generative fidelity, measuring distribution-level realism via Fréchet Video Distance (FVD-CST); (ii) structural similarity, evaluating voxel-level and spatial agreement (L1, PSNR, SSIM); and (iii) downstream biomarker evaluation, measuring the forecast change of central subfield thickness (CST) and of an OCT-derived estimate of best-corrected visual acuity (BCVA) with frozen, eye-level cross-validated regressors, and comparing it with the recorded clinical labels. All comparisons are paired at the eye level with hierarchical bootstrap confidence intervals, complemented by control experiments and a five-fold cross-validation over all 52 time-annotated eyes. Experiments on the longitudinal OLIVES clinical trial cohort show that our framework achieves the highest structural similarity in every test eye (SSIM 0.657 vs. 0.543–0.623 for the comparators) and the highest point estimates of CST change tracking (Δ-*r* = 0.880, Δ-MAE = 12.7 μm). Across the 52-eye cross-validation, it improves PSNR over an identity copy by 1.27 dB (95% CI 0.93–1.57) and reduces the CST change error by 10.5 μm (95% CI 3.1–19.4).

The primary contributions of this work are summarized as follows:

1. We identify the empirical failure mode of direct volumetric generation in interventional longitudinal retinal imaging, establishing that stationary anatomical dominance causes regression-to-the-mean and biomarker decoupling.
2. We propose a two-stage framework that explicitly decouples baseline ocular anatomy from treatment-associated dynamics, discretizing localized change via FSQ for patient-specific 3D forecasting.
3. We evaluate the forecasts with eye-level statistics, control experiments, clinical labels and a 52-eye cross-validation, showing consistent gains in structural fidelity and CST change tracking over generative baselines, and we characterize where the forecasts help (long horizons, marked responses) and where they fail (stable eyes, short intervals).

## 2. Materials and Methods

### 2.1. Longitudinal Clinical Trial Cohort and Preprocessing

This study utilizes the public OLIVES (Ophthalmic Labels for Investigating Visual Eye Semantics) dataset [45], which aggregates longitudinal spectral-domain OCT (SD-OCT) volumes from multi-center anti-VEGF clinical trials in diabetic retinal disease, specifically diabetic retinopathy (DR) and diabetic macular edema (DME). The released cohort comprises 96 eyes imaged across 1591 visits, together with per-visit clinical labels including best-corrected visual acuity (BCVA, ETDRS letters) and central subfield thickness (CST, μm).

As detailed in Table 1, the cohort is structured into four treatment regimens: the PRIME trial (40 eyes), whose visit records encode exact study weeks from W0 to W104 (up to 21 longitudinal time points per eye), and three TREX-DME regimens—Monthly (12 eyes), GILA (22 eyes), and TREX (22 eyes)—for which only ordinal visit indices are provided. PRIME (NCT03531294) enrolled 40 eyes with treatment-naïve DR (Diabetic Retinopathy Severity Scale [DRSS] levels 47A–71A) without center-involved DME; eyes with a central subfield thickness above 320 μm or with central DME causing visual loss were excluded [5,46]. All PRIME eyes received monthly intravitreal aflibercept 2.0 mg until a DRSS improvement of at least two steps; eyes were randomized 1:1 to DRSS-guided or panretinal leakage index (PLI)-guided re-treatment: monthly injections resumed after a one-step DRSS worsening from the best level achieved (DRSS arm) or when the PLI rose to at least 50% of the difference between the baseline and threshold PLI (PLI arm), and stopped once the best DRSS level or the threshold PLI was regained. PRIME visits took place every 4 weeks through week 52 and every 8 weeks through week 104. TREX-DME randomized 150 eyes with center-involved DME 1:2:2 to monthly dosing, treat-and-extend (TREX), or treat-and-extend with angiography-guided macular laser (GILA), all with ranibizumab 0.3 mg [47]; the Monthly arm received an injection every 4 weeks. Monthly visits are therefore assigned nominal timestamps of 4×(n−1) weeks, which may deviate from the actual dates within the protocol visit window; in contrast, GILA and TREX follow individualized treat-and-extend intervals whose visit weeks are only partially recorded in the released spreadsheets and are not linked to the released volumes by an identifier, so these arms are excluded from time-conditioned forecasting. The resulting time-annotated subset contains 52 eyes and 961 visits, with inter-visit intervals spanning 4 to 104 weeks (median 28 weeks; 60.1% of derived pairs carry exact PRIME intervals). Follow-up intervals are discretized into 26 four-week bins, b=⌊(Δt−1)/4⌋, covering weeks 1–104.

For PRIME, OLIVES records the injection status of the study eye at each visit, and 88.1% of the PRIME pairs (95.2% of the PRIME test pairs) span at least one recorded injection. Per-visit injection records are not released for TREX-DME; the Monthly arm received ranibizumab every 4 weeks by protocol. Each pair is therefore a follow-up interval within an active anti-VEGF treatment protocol; the injection history within the interval is not used as a model input. Accordingly, we model treatment-associatedanatomical change in observational trial data; the observed change may also reflect the natural disease course and other clinical factors, and we do not estimate causal treatment effects.

**Volume Processing and Longitudinal Pairing:** Each study visit is formatted as a 3D volume with dimensions of D×H×W=46×112×112 voxels. Raw voxel intensities in [0,255] are normalized to [−1,1] via a fixed affine scaling:
  (1) $$x=x_{\mathrm{raw}}\times \left(\frac{2}{255}\right)-1\;\in \left[-1,1\right],$$
  which aligns with the hyperbolic tangent (tanh) activation range of the decoder while preserving physical scanner calibration across longitudinal visits. Longitudinal visit pairs are assembled chronologically within each unique eye: every strictly forward visit combination forms one candidate pre- and post-treatment $\left(X_{\mathrm{pre}},X_{\mathrm{post}}\right)$ pair, yielding 13,956 pairs across the full 96-eye cohort. Enumerating all forward combinations covers follow-up horizons from 4 to 104 weeks, which the interval conditioning requires. Pairs spanning intermediate visits reflect the cumulative change over the interval; intermediate visits are not used as model inputs. To strictly prevent patient overlap and data leakage, splits are assigned at the eye level (78 training, 9 validation, and 9 test eyes) and stratified by treatment regimen, yielding 11,462 training, 1203 validation, and 1291 test pairs. Time-conditioned forecasting experiments restrict this manifest to the time-annotated subset, comprising 9227 pairs from 52 eyes partitioned at the eye level into 42 training eyes (7641 pairs), 5 validation eyes (848 pairs), and 5 held-out test eyes (738 pairs). Pairs from the same eye are highly correlated, so the number of independent units is the number of eyes, not the number of pairs; all statistical inference in this study therefore resamples or permutes eyes (Section 2.5), and we additionally report a five-fold eye-level cross-validation over all 52 eyes (Section 2.5).

### 2.2. Stage 1: Retinal Response Tokenizer

The primary objective of Stage 1 is to construct a compact, structured representation of longitudinal anatomical change while anchoring invariant ocular morphology. We address this through an asymmetrical bottleneck architecture, as illustrated in Figure 1 (left).

#### 2.2.1. Factorized 3D Spatial-Depth Encoding

Clinical SD-OCT imaging is physically anisotropic: the in-plane resolution within a single B-scan (transverse and axial) is significantly denser than the sparse sampling interval between consecutive B-scans along the depth axis. Standard isotropic 3D convolutions fail to model these disparate physical scales. We employ a 3D patch embedding layer to project the input volume $X\in R^{1\times D\times H\times W}$ into a token grid:

(2) $$Z_{0}\in R^{D^{\prime }\times H^{\prime }\times W^{\prime }\times C},$$

where *C* denotes the hidden embedding dimension, and $\left(D^{\prime },H^{\prime },W^{\prime }\right)$ represent the downsampled depth, height, and width token dimensions. With patch size (2,8,8) and C=512, the grid is 23×14×14×512. To capture long-range layer correlations efficiently, the encoder consists of stacked factorized transformer blocks inspired by video architectures [42]. Each block sequentially applies:

1. **Spatial Self-Attention:** Computes multi-head self-attention across transverse tokens independently for each depth slice, capturing intraretinal cyst boundaries and layer interfaces.
2. **Depth Self-Attention:** Computes self-attention across depth tokens independently for each spatial coordinate, capturing volumetric fluid pocket extension across parallel B-scans.

Learned separable positional encodings are added along the depth, height, and width axes, yielding continuous latent feature grids $z_{\mathrm{pre}}=E\left(X_{\mathrm{pre}}\right)$ and $z_{\mathrm{post}}=E\left(X_{\mathrm{post}}\right)$ (Continuous Latents, denoted by cyan slabs in Figure 1).

#### 2.2.2. Latent Residual Extraction and Quantization

Because the surrounding retinal boundary, scleral curvature, and choroidal architecture are largely conserved across visits (quantified in Section 2.5), monolithic volumetric representation spends representational capacity on stationary background anatomy. We isolate the longitudinal change through a latent residual. Both latent grids are mapped by a shared bottleneck ϕ—a linear projection to d=6 channels followed by two 3×3 convolutions (the first with stride 2) applied to each depth slice—onto an action grid of 23×7×7 positions, and the residual

(3) $$\Delta z=\phi \left(z_{\mathrm{post}}\right)-\phi \left(z_{\mathrm{pre}}\right)\in R^{N\times d},\;N=23\times 7\times 7=1127,$$

has unbounded coordinates $v\in R^{d}$ at each position. This residual formulation is designed to isolate localized change from stationary background anatomy.

We discretize v using Finite Scalar Quantization (FSQ) [43]. Unlike traditional Vector Quantization (VQ-VAE) [48,49] or residual vector quantization (RQ-VAE) [50], which rely on learned codebook lookups prone to codebook collapse and index unreliability in small medical cohorts, FSQ applies a fixed, lookup-free rounding onto predefined integer levels $L=(L_{1},\ldots ,L_{d})$. The quantized coordinate ${\hat{v}}_{i}$ along channel *i* is defined as:

(4) $${\hat{v}}_{i}=\frac{1}{\left\lfloor L_{i}/2\right\rfloor }\;\mathrm{round}\left(h_{i}\tanh\left(v_{i}+\mathrm{artanh}\left(o_{i}/h_{i}\right)\right)-o_{i}\right),\;h_{i}=\frac{L_{i}-1}{2},$$

where $o_{i}=1/2$ for even $L_{i}$ and $o_{i}=0$ for odd $L_{i}$, so that channel *i* takes exactly $L_{i}$ values [43]. This formulation generates an exact, non-collapsible discrete codebook of size $K=\prod_{i=1}^{d}L_{i}$ unique states. We use L=(8,8,8,5,5,5), i.e., K=64,000 states per position; this configuration was fixed a priori to favor reconstruction fidelity. Straight-through gradient estimation is employed during backpropagation. The quantized coordinate vectors are re-projected to feature dimension *C*, yielding the discrete action sequence:

(5) $$a={\left\{\Delta z_{k}^{q}\right\}}_{k=1}^{N},\;\Delta z_{k}^{q}\in R^{C},$$

where *N* denotes the total number of localized action tokens across the spatial-depth grid (Discrete Tokens, denoted by segmented blue blocks in Figure 1).

#### 2.2.3. Baseline-Anchored Conditional 3D Decoding

To ensure that the reconstructed post-treatment scan preserves individual retinal morphology, the decoder does not synthesize the volume in isolation. Instead, the continuous pre-treatment baseline feature tokens $z_{\mathrm{pre}}$ (Continuous Latents) serve as queries in multi-head cross-attention blocks, where the quantized action tokens $\Delta z^{q}$ (Discrete Tokens) act as keys and values:

(6) $$z_{\mathrm{cond}}=\mathrm{CrossAttention}\left(Q=z_{\mathrm{pre}},K=\Delta z^{q},V=\Delta z^{q}\right).$$

Factorized 3D transformer blocks refine $z_{\mathrm{cond}}$, followed by 3D transposed convolutions to reverse patchification. A final hyperbolic tangent activation yields the reconstructed 3D volume ${\hat{X}}_{\mathrm{post}}=D\left(z_{\mathrm{pre}},\Delta z^{q}\right)\in {\left[-1,1\right]}^{1\times D\times H\times W}$.

Stage 1 is optimized end-to-end via a composite volumetric loss function:

(7) $$L_{\mathrm{Stage}1}=L_{1}\left({\hat{X}}_{\mathrm{post}},X_{\mathrm{post}}\right)+\lambda_{\mathrm{grad}}L_{\mathrm{grad}}\left({\hat{X}}_{\mathrm{post}},X_{\mathrm{post}}\right)+\lambda_{\mathrm{lpips}}L_{\mathrm{LPIPS}}\left({\hat{X}}_{\mathrm{post}},X_{\mathrm{post}}\right),$$

where $L_{1}$ enforces voxel-level absolute fidelity, $L_{\mathrm{grad}}$ computes multi-axial 3D finite differences to maintain sharp retinal boundary gradients, and $L_{\mathrm{LPIPS}}$ evaluates perceptual layer stratification using a slice-wise feature extractor [51]. This reconstruction objective provides the Stage 1 supervision signal (indicated by the red dashed line in Figure 1, left).

### 2.3. Stage 2: Prognostic Action Generator

In clinical inference, the post-treatment scan $X_{\mathrm{post}}$ is unobserved. Stage 2 forecasts the patient-specific discrete action sequence $\hat{a}$ (representing $\Delta z^{q}$) conditioned on the baseline observation $X_{\mathrm{pre}}$ (denoted $X_{0}$) and the clinical condition slot (c,Δt)—encompassing the scheduled follow-up interval Δt (weeks) and baseline clinical covariates *c* (including CST, BCVA, and treatment protocol)—as illustrated in Figure 1 (right). Following formulations in latent action sequence modeling [44], the forecasting task operates over the structured discrete vocabulary of localized actions.

#### 2.3.1. Conditioning and Action Transformer

The baseline volume $X_{\mathrm{pre}}$ is processed by the frozen Stage 1 spatial encoder (marked with a snowflake icon in Figure 1; 25.6 M parameters) to produce continuous anatomical context tokens $z_{\mathrm{pre}}\in R^{23\times 14\times 14\times 512}$, which are linearly projected to the model width. Two condition tokens are prepended: an interval token, obtained by a sinusoidal embedding of the interval bin *b* (26 four-week bins, Section 2.1), and a covariate token, obtained by a gated MLP applied to $c=[{\mathrm{CST}}_{\mathrm{pre}},{\mathrm{BCVA}}_{\mathrm{pre}},e_{\mathrm{arm}}]$, where $e_{\mathrm{arm}}$ is a one-hot encoding of the regimen (Monthly or PRIME). The sequence of 2+23×14×14 tokens is processed by an Action Transformer with 6 layers, 8 attention heads and hidden size 512 (20.9 M trainable parameters), using a causal (left-to-right) attention mask over a depth-slice-first raster order and three-dimensional multimodal rotary position embeddings [52]. The output features of each depth slice are average-pooled from 14×14 to the 7×7 action grid, so that all N=1127 action positions are predicted in a single forward pass.

#### 2.3.2. Factorized Token Prediction and Training

Each action token is an FSQ code with six digits, (8,8,8,5,5,5). Learning a categorical distribution over all 64,000 states per position is not feasible with 42 training eyes. The Action Transformer therefore predicts only the three coarse digits (8×8×8=512 states per position) with three factorized linear heads, whose logits are added to an empirical per-position log-prior estimated on the training pairs. The output heads are initialized at zero, so that the model starts from the positional prior and learns patient-specific deviations from it. Training minimizes the cross-entropy between the predicted and ground-truth coarse digits of the training pairs:

(8) $$L_{\mathrm{Stage}2}=\sum_{j=1}^{3}L_{\mathrm{CE}}\left({\hat{a}}^{\left(j\right)},a^{(j),\mathrm{GT}}\right).$$

The three fine digits (5×5×5=125 states) are not learned: at inference they are sampled from an empirical conditional table p(fine∣position,coarse) estimated on the training pairs. One sample is drawn per pair.

During inference, the predicted discrete action tokens $\hat{a}\equiv \Delta z_{\mathrm{pred}}^{q}$ (Discrete Tokens, blue segmented blocks) and continuous baseline latents $z_{\mathrm{pre}}$ (Continuous Latents, cyan slabs) are routed to the frozen Stage 1 De-Tokenizer (Conditional Decoder D, marked with a snowflake icon in Figure 1), which applies multi-head cross-attention and transposed convolutions to synthesize the final forecasted 3D-OCT volume:

(9) $${\hat{X}}_{\mathrm{post}}=D\left(z_{\mathrm{pre}},\hat{a}\right),$$

binding predicted changes to the patient’s baseline ocular morphology.

### 2.4. Downstream Regressors

CST and BCVA cannot be read directly from a generated volume, so they are measured by two regressors initialized from the publicly released RETFound OCT weights [53] and fine-tuned on OLIVES volumes with recorded labels (CST: 670 volumes from 73 eyes; BCVA: 706 volumes from 73 eyes). Training used five-fold eye-level cross-validation; the last 24 transformer blocks were fine-tuned (learning rates 10^−5^ for the backbone and 10^−3^ for the regression head). At evaluation, every volume—baseline, real follow-up, and forecast—is scored by the fold model whose training data did not contain that eye, so no image or label of a test eye contributes to the model that scores it; eyes outside the 73 labeled eyes are scored by the mean of the five fold models. The regressors were developed independently of the generative models: their hyper-parameters were chosen only on the pooled out-of-fold agreement with the clinical labels, and all methods are scored with the same final regressors. Pooled out-of-fold agreement with the clinical labels is r=0.958 (MAE 17.8 μm) for CST and r=0.577 (MAE 7.0 letters) for BCVA (Supplementary Table S2). Because BCVA is a functional endpoint with only moderate structure–function correlation, we treat the regressor output as an OCT-derived BCVA estimate and use it as a secondary, indirect endpoint.

### 2.5. Evaluation Dimensions and Statistical Analysis

Prognostic 3D-OCT synthesis is benchmarked across three complementary dimensions:

- **Distribution Fidelity:** We compute Fréchet Video Distance (FVD−CST) using deep feature representations from the ophthalmic foundation model RETFound [53] to quantify distributional realism against ground-truth volume cohorts.
- **Voxel and Structural Fidelity:** Mean absolute error (L1), Peak Signal-to-Noise Ratio (PSNR), and Structural Similarity Index (SSIM) are averaged across volumetric B-scans to measure spatial reconstruction and anatomical contour preservation.
- **Downstream Biomarker Evaluation:** Because stationary ocular anatomy can inflate voxel-level metrics, we evaluate the forecast change of CST and of the OCT-derived BCVA estimate. With the regressor *f*, the forecast change is $\hat{\Delta }=f\left({\hat{X}}_{\mathrm{post}}\right)-f\left(X_{\mathrm{pre}}\right)$ and the reference change is $\Delta =f\left(X_{\mathrm{post}}\right)-f\left(X_{\mathrm{pre}}\right)$; we report the change error Δ-MAE $=|\hat{\Delta }-\Delta |$, the Pearson correlation Δ-*r* between $\hat{\Delta }$ and Δ, and the agreement in the direction of change. We additionally evaluate $\hat{\Delta }$ against the change of the recorded clinical labels. CST errors are referenced to the reproducibility of OCT CST measurements in DME (median absolute difference between replicate scans, 7 μm; half-width of the 95% CI for a change, 38 μm) [1,54], and BCVA errors to a ±7-letter tolerance, which lies between the 5- and 10-letter limits of ETDRS test–retest variability [55].

**Statistical analysis.** Pairs from the same eye are not independent. Confidence intervals (95%) are therefore obtained with a hierarchical bootstrap [56]: eyes are resampled with replacement, then pairs within each resampled eye (2000 replicates). Comparisons between methods are paired on the same held-out eyes: for each metric, we report the difference to our method with its bootstrap CI (both methods evaluated on the same replicates), the number of eyes in which our method is better, and an eye-level sign-flip test on per-eye mean differences (exact enumeration for up to 16 eyes, 20,000 Monte Carlo draws otherwise). With five test eyes, the smallest attainable two-sided exact *p* is 0.0625, so test split conclusions rest on the bootstrap CIs. FVD-CST is a distribution-level metric without per-pair values and is reported as a point estimate.
**Controls and non-imaging baselines.** To test whether forecasts depend on patient-specific inputs, we keep the decoder and all priors fixed and change only the source of the drift tokens: (a) a no-conditioning control draws the coarse digits from the population positional prior, without image, interval or covariates; (b) a shuffled-eye control uses drift tokens generated for a different eye. Non-imaging baselines regress the CST or BCVA change on the same clinical covariates (baseline CST, baseline BCVA, interval, regimen) with an interval-wise mean, ridge regression, or gradient boosting; their hyper-parameters were chosen on the validation split.
**Stratified and sensitivity analyses.** Results are stratified by follow-up horizon (≤12, 13–26, 27–52 and >52 weeks) and by the magnitude of the clinically measured CST change (<10 μm, i.e., close to the test–retest difference of OCT CST measurements; 10–25 μm; ≥25 μm). As sensitivity analyses of the pairing strategy, we repeat the evaluation on consecutive-visit pairs only (no intermediate visits) and on baseline-anchored pairs (first visit to each later visit).
**Cross-validation.** To increase the number of independent test units beyond five eyes, we performed a five-fold eye-level cross-validation of the forecasting stage over all 52 time-annotated eyes. In each fold, the Action Transformer, the positional prior and the fine-digit table were re-estimated on the training folds only, with the fixed recipe of the main model and no per-fold selection (final-epoch checkpoint). The Stage 1 tokenizer was kept fixed; results for the 10 eyes that the tokenizer had not seen are given in Supplementary Table S5.
**Stationary anatomy.** To quantify the fraction of anatomy that does not change, voxel-wise change $|X_{\mathrm{post}}-X_{\mathrm{pre}}|$ was computed for all 9227 time-annotated pairs after light 3D Gaussian smoothing (σ=1 voxel) to suppress speckle. A noise floor *τ* was defined as the 95th percentile of this change within retinal tissue in 260 anatomically unchanged reference pairs (interval ≤4 weeks, |ΔCST| ≤ 2 μm), so that *τ* includes acquisition noise and inter-visit misalignment (volumes are not registered, which makes the estimate conservative). Retinal tissue was defined by an Otsu threshold. A voxel is counted as static if its change is below *τ*; sensitivity is assessed with *τ* at the 90th and 99th percentiles.

### 2.6. Implementation Details

The Stage 1 tokenizer uses hidden dimension C=512, patch size (2,8,8), eight spatial and eight depth attention layers with 8 heads, and FSQ levels (8,8,8,5,5,5) on the 23×7×7 action grid. The Action Transformer (6 layers, 8 heads, hidden size 512; 20.9 M trainable parameters) was trained with AdamW (β=(0.9,0.95), learning rate 10^−4^, weight decay 0.05), dropout 0.2, paired horizontal flipping (p=0.5), global batch size 96, and bf16 mixed precision for 10 epochs (approximately 3 min on six NVIDIA L20X GPUs). All models were implemented in Python 3.10 with PyTorch 2.8.0 (CUDA 12.8). The reported checkpoint is one of the checkpoints retained by the validation-loss callback (top-3; validation losses within 0.02 nats). The FSQ configuration was fixed a priori; the codebook size and generation paradigm ablations (Section 3.5) were run after the main model had been fixed and were not used to select it. All settings are listed in Supplementary Table S8.

## 3. Results

### 3.1. Benchmark Comparison on Held-Out Clinical Cohort

We benchmark our proposed framework against established generative paradigms on the held-out test cohort of 738 longitudinal 3D volume pairs from five independent eyes: (1) Identity Copy ($X_{0}$), serving as the zero-operation static baseline; (2) CycleGAN [37], representing adversarial image-to-image translation adapted for volumetric forecasting; (3) Palette [39], representing conditional pixel space diffusion with a 3D U-Net backbone; (4) I2I-DiT [40], representing scalable Diffusion Transformers operating on continuous volumetric latents; and (5) VQGAN [49], representing discrete autoregressive generation. All models were trained under identical hardware protocols and evaluated on the same held-out test pairs. Implementation details of our model are given in Section 2.6.

As summarized in Table 2 and Table 3 (complete metrics with confidence intervals for all methods and controls in Supplementary Table S1), the benchmark reveals distinct characteristics across the three evaluated dimensions:

1. **Generative Fidelity vs. Structural Discordance in Adversarial Modeling:** Adversarial CycleGAN achieves the lowest FVD-CST (5.0) and sharp slice textures (PSNR 22.50 dB). However, its adversarial loss prioritizes local patch realism over longitudinal change: its CST change correlation is limited (Δ-r=0.465), and its predicted OCT-derived BCVA changes correlate negatively with the reference (Δ-r=−0.166).
2. **Continuous Mean-Regression in Diffusion Baselines:** Monolithic continuous diffusion models (Palette, I2I-DiT) achieve moderate change correlations (Δ-r=0.777−0.798), but suffer from structural blurring (SSIM 0.547–0.567), falling below the static Identity baseline (SSIM 0.604). Most retinal tissue does not change between visits (a median of 94% of tissue voxels change by less than the inter-visit noise floor; Section 3.6), so the zero-operation Identity copy conserves these regions, whereas unconstrained diffusion regenerates all voxels, introducing textural fluctuations that penalize SSIM.
3. **Discrete Codebook Instability in Autoregressive Baselines:** Standard autoregressive VQGAN achieves an FVD-CST of 14.8, but exhibits discrete boundary quantization artifacts across 3D depth, resulting in higher change error (CST Δ-MAE = 19.3 μm) and lower structural fidelity (SSIM = 0.543).
4. **Decoupled Latent Action Forecasting:** By anchoring stationary baseline anatomy and confining generation to quantized action tokens, our framework obtains the best point estimates for all structural similarity metrics (L1 = 0.0965, PSNR = 22.55 dB, SSIM = 0.657) and for the downstream change metrics: CST Δ-*r* = 0.880 (Δ-MAE = 12.7 μm) and BCVA Δ-r=0.773 (Δ-MAE = 3.03 letters).

**Paired eye-level comparison.** With only five test eyes, several intervals in Table 2 are wide, and differences must be judged on paired comparisons (Table 3). Structural similarity is robust even at this sample size: our method achieves the highest SSIM in all five eyes against every comparator, and the 95% CIs of its SSIM advantage exclude zero for all of them (+0.053 [0.026, 0.073] over the identity copy, +0.034 [0.019, 0.043] over CycleGAN, and +0.090 to +0.114 over the diffusion and VQGAN baselines). The PSNR advantage is significant against Palette (+1.90 dB [1.30, 2.24]), I2I-DiT (+1.84 dB [1.16, 2.74]) and VQGAN (+1.43 dB [0.82, 1.73]), all in five of five eyes, but not against the identity copy or CycleGAN. For the CST change error, the point estimates favor our method against every comparator, but all CIs include zero on the five-eye test split; the 52-eye cross-validation below provides the more powerful test.

**Figure 2:**
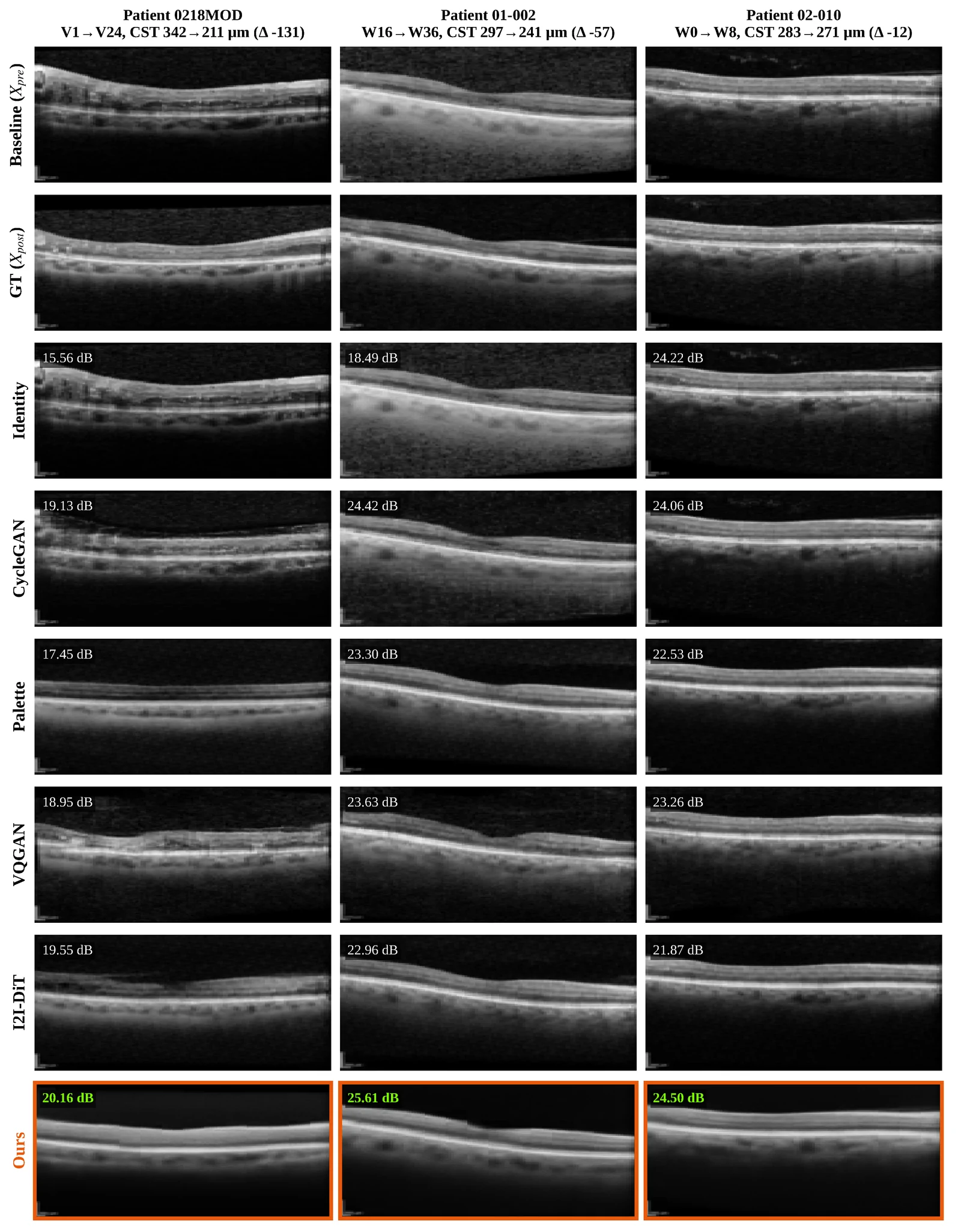
Qualitative comparison of longitudinal 3D-OCT forecasting across representative patients. Three clinical cases from the held-out test cohort illustrate distinct pathological presentations: central intraretinal cystoid edema (Case 1), subretinal fluid detachment (Case 2), and multi-focal laminar microcysts (Case 3). From top to bottom: pre-treatment baseline ($X_{\mathrm{pre}}$), ground-truth post-treatment scan ($X_{\mathrm{post}}$), static identity copy, comparative baselines, and the proposed decoupled framework (bottom row, framed in orange with PSNR values in upper left). Our method follows the fluid resolution and the foveal depression of the real follow-up while preserving the baseline layer structure. Rule-based failure cases are shown in Supplementary Figure S3.

Qualitative comparisons across representative treatment cases are presented in Figure 2. In Case 1 (large central intraretinal cystoid edema), CycleGAN fails to resolve the central pocket, retaining hyper-reflective cyst remnants. Palette and I2I-DiT reduce cyst volume but leave an over-smoothed, flattened foveal pit. Our method reproduces the fluid clearance and the native foveal dip of the ground-truth. In Case 2 (subretinal fluid detachment), our framework reconstructs re-apposition of the neurosensory retina to the RPE layer without boundary artifacts. In Case 3 (multi-focal microcysts in inner nuclear layers), diffusion baselines diffuse cyst fluid into adjacent parenchyma, whereas our discrete action generator preserves individual layer stratification.

**Figure 3:**
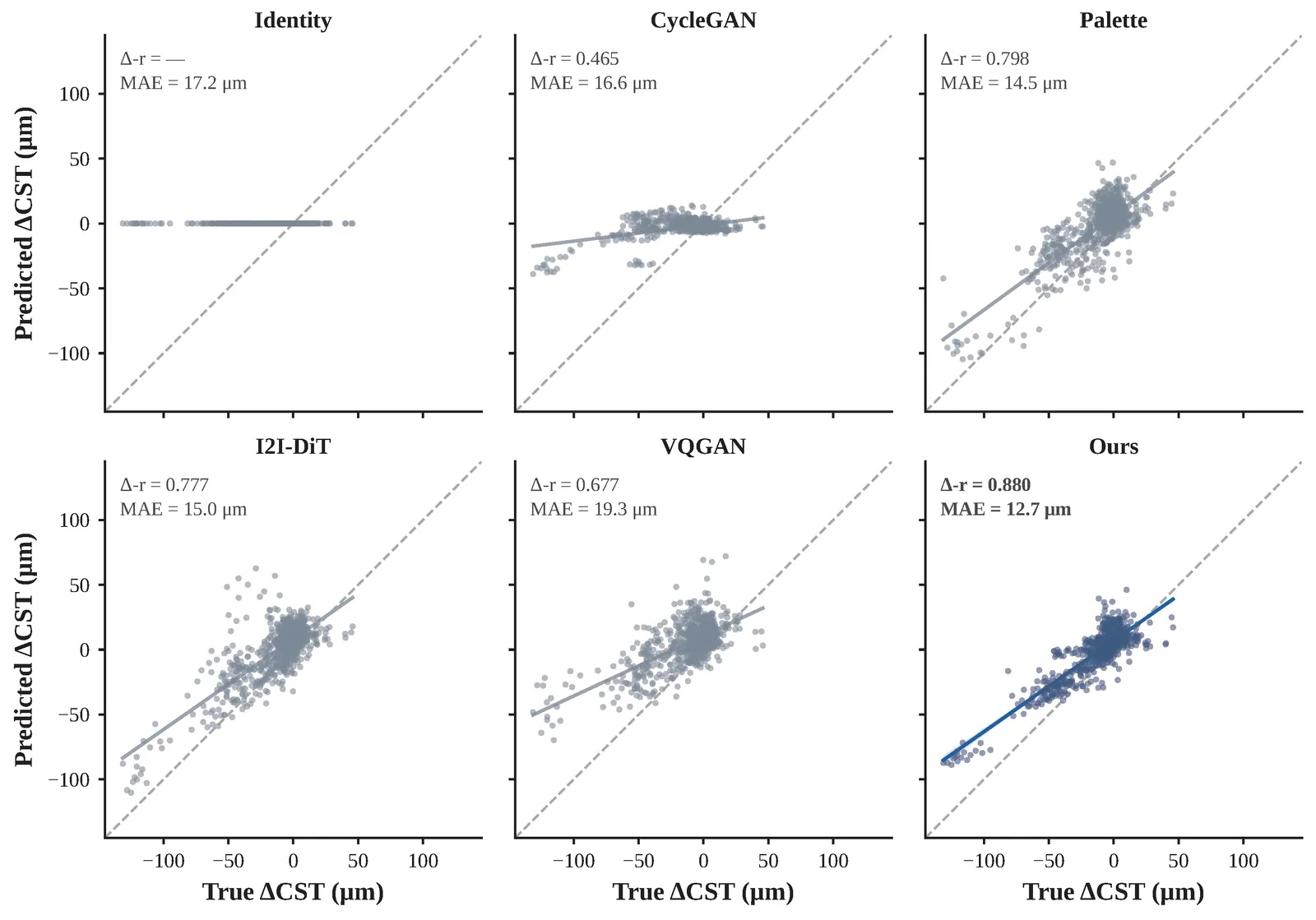
Downstream anatomical biomarker benchmark across evaluated forecasting models. Forecast CST change ($\Delta {\mathrm{CST}}_{\mathrm{pred}}$) versus the change measured by the same frozen regressor on the real follow-up scan ($\Delta {\mathrm{CST}}_{\mathrm{GT}}$) for all 738 test pairs (five eyes). Dashed lines: identity (y=x); solid lines: linear fits; insets: Δ-*r* and MAE (μm). Eye-level CIs are given in Table 2.

Quantitative distribution analysis of the downstream anatomical biomarker is depicted in Figure 3. Scatter plots demonstrate that CycleGAN exhibits wide heteroscedastic spread, with numerous predictions severely under- or over-estimating fluid resolution. While 3D Palette and I2I-DiT exhibit improved clustering along the diagonal, they demonstrate compression toward zero change for large fluid reductions (>150 μm). Our framework maintains linear concordance across both mild and severe edema cases (r=0.880), with a mean error of 12.7 μm; for reference, the 95% limit for a real change between repeated OCT CST measurements is 38 μm [54].

**Figure 4:**
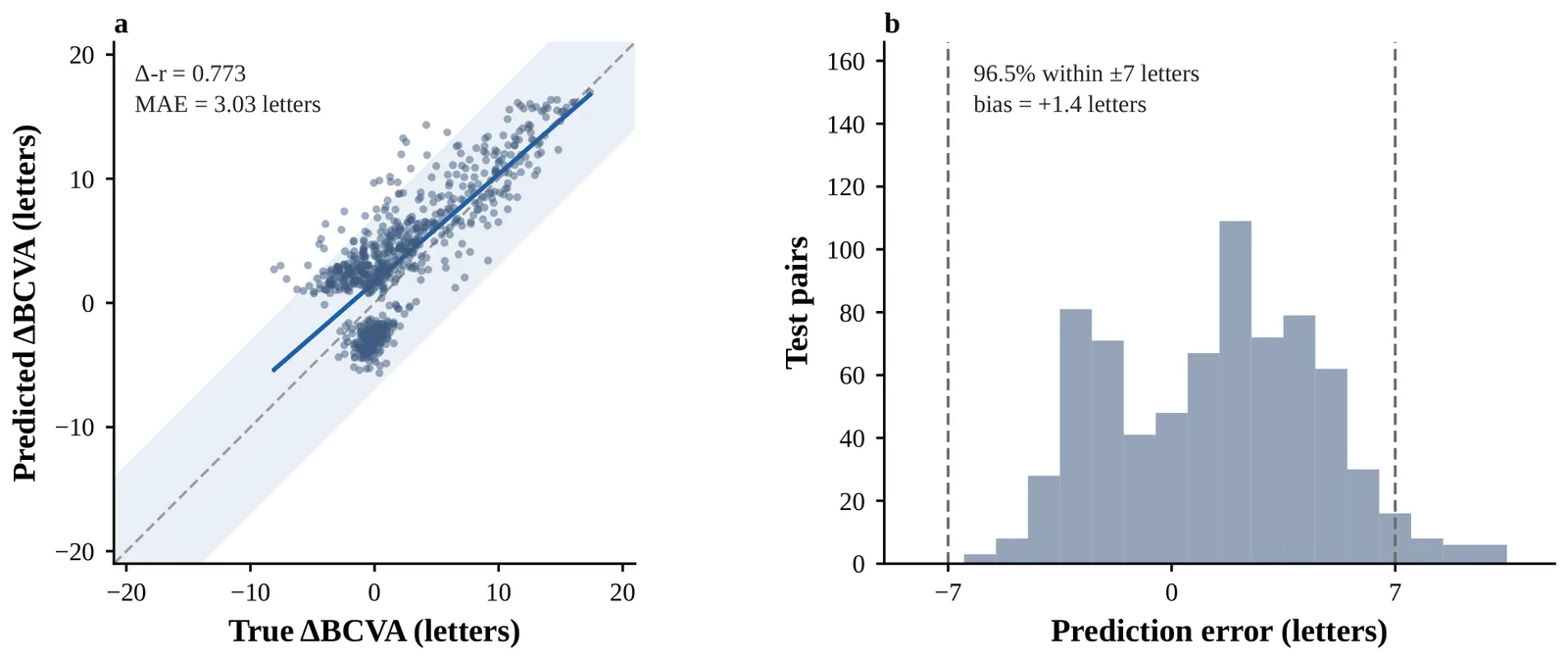
OCT-derived BCVA estimate on the held-out test cohort. (**a**) Forecast versus reference change of the OCT-derived BCVA estimate ($\Delta {\mathrm{BCVA}}_{\mathrm{pred}}$ vs. $\Delta {\mathrm{BCVA}}_{\mathrm{GT}}$, ETDRS letters) for our method; dashed line: y=x; shaded: confidence envelope. (**b**) Histogram of the differences for all 738 test pairs; dashed lines mark the ±7-letter tolerance (Section 2.5). Both quantities are regressor outputs, so this does not show prediction of clinical visual acuity.

The OCT-derived BCVA estimate is evaluated in Figure 4. Forecast and reference estimates agree closely (Δ-r=0.773, Δ-MAE = 3.03 letters), and 96.5% of the differences lie within ±7 letters with a bias of +1.4 letters. Because both quantities are outputs of the same OCT-based regressor, whose own agreement with the clinical BCVA change is limited (Δ-r=0.40 on the test split; Section 3.2), this result shows that the forecasts preserve the image features the regressor relies on, not that clinical visual acuity is predicted.

### 3.2. Change-Focused Evaluation and Patient Specificity

To test whether the forecasts carry patient-specific information rather than a generic change pattern, Table 4 compares our method with the two controls, which share our decoder and priors, and with non-imaging baselines. Both controls are consistently worse than our forecasts: our method improves PSNR by +0.15 dB [0.03, 0.25] over the no-conditioning control and by +0.22 dB [0.10, 0.32] over the shuffled-eye control, in five of five eyes each, and lowers the BCVA change error by +0.42 [0.05, 0.73] and +0.49 [0.05, 0.83] letters (Table 3). The CST change correlation drops from 0.88 to 0.69 and 0.61, and the CST change error rises by 4.2 and 4.4 μm (the latter with a CI excluding zero, [0.11, 8.39]). Non-imaging baselines that use the same clinical covariates track the CST change far less well (Δ-*r* 0.29–0.55).

Against the recorded clinical CST labels, change tracking is preserved: our method reaches Δ-r=0.65, equal to the diffusion baselines (0.65) and higher than VQGAN (0.47), CycleGAN (0.33) and both controls (0.40 and 0.29); the regressor applied to the real follow-up scans, i.e., the ceiling of any image-based forecast, reaches 0.77 (Supplementary Table S4). For BCVA, the regressor’s own ceiling against the clinical labels is low (Δ-r=0.40), and no image-based forecast outperforms the identity copy or the non-imaging baselines against the BCVA labels; we therefore do not claim prediction of clinical visual acuity.

### 3.3. Cross-Validation over 52 Eyes

**Figure 5:**
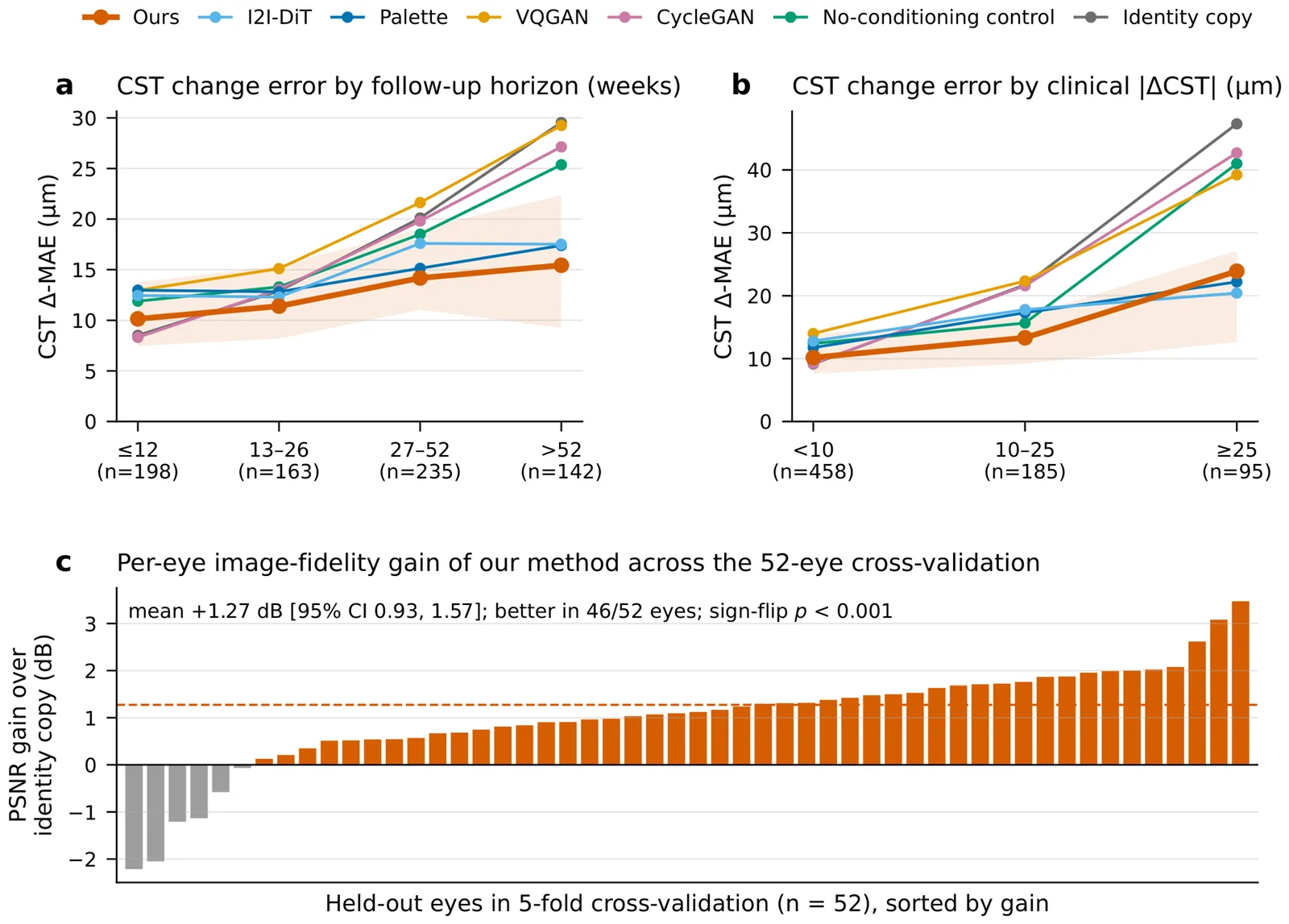
Where the forecasts help. (**a**) CST change error by follow-up horizon and (**b**) by the magnitude of the clinically measured CST change on the test split (frozen regressor; shaded: eye-level 95% CI for our method). The identity copy is accurate when little changes, whereas our forecasts keep the error low for long horizons and marked responses. (**c**) Per-eye PSNR gain over the identity copy for all 52 held-out eyes of the five-fold cross-validation.

The five-fold eye-level cross-validation holds out every time-annotated eye once from the forecasting stage (Table 5, Figure 5c). Across the 52 held-out eyes, our method improves PSNR over the identity copy by +1.27 dB [0.93, 1.57] (better in 46 of 52 eyes, p<0.001) and lowers the CST change error by 10.5 μm [3.1, 19.4] (p=0.009); against the clinical CST labels, the reduction is 8.6 μm [0.3, 18.7]. The CST change correlation is Δ-r=0.84 [0.69, 0.90], with a much narrower interval than on the five-eye test split. The advantage over the no-conditioning control also holds in this larger evaluation (PSNR +0.13 dB [0.03, 0.22], p=0.004; CST Δ-*r* +0.048 [0.022, 0.081]).

### 3.4. Follow-Up Horizon and Magnitude of Response

Table 6 and Figure 5a,b stratify the test results. The advantage of our method grows with the horizon (27–52 weeks: 14.2 vs. 20.1 μm for the identity copy; >52 weeks: 15.4 vs. 29.5 μm), and its CST change correlation rises from 0.60 (≤12 weeks) to 0.96 (>52 weeks). For marked changes (≥25 μm), its error is 23.9 μm, compared with 47.3 μm for the identity copy, 41.0 μm for the no-conditioning control, and 44.3 μm for the best non-imaging baseline. The same pattern holds against the clinical labels (Supplementary Table S10), and on baseline-anchored pairs our method shows the strongest change tracking (Δ-r=0.97; Supplementary Table S3). When little changes, i.e., for intervals of at most 12 weeks or clinically stable pairs (|ΔCST| < 10 μm), the identity copy is accurate (8.5 vs. 10.1 μm and 9.1 vs. 10.2 μm).

The benefit at long horizons derives mainly from the baseline image. When only the interval token was varied for a fixed baseline (80 test baselines, intervals from 4 to 104 weeks), the predicted CST change varied little (+1.7 to +3.4 μm on average), whereas the observed change grows with the interval. The model should therefore not be used to compare alternative follow-up intervals.

### 3.5. Ablation Study and Architectural Analysis

To assess the contribution of each component, we conducted ablation experiments across three design dimensions, reported in Table 7. All ablations were run after the main model had been fixed and were not used to select it.

**Residual Decoupling Strategy:** We examine replacing the latent residual target Δ*z* with direct monolithic volumetric synthesis in pixel space (Direct Pixel Synthesis). As shown in Table 7A, forcing the network to synthesize future volumes from scratch causes a drop in structural fidelity: SSIM plunges from 0.657 to 0.556, while CST thickness error increases by 28.3% (from 12.7 μm to 16.3 μm, with correlation dropping to 0.671). This validates our foundational premise: in ocular pharmacotherapy, isolating the longitudinal residual prevents model capacity from being wasted on static tissue.
**Stage 2 Generation Paradigm:** All three variants share the same Stage 1 tokenizer and baseline-query cross-attention decoder and differ in how the action tokens are generated: (1) autoregressive decoding, token by token; (2) MaskGIT-style iterative unmasking [57]; and (3) our single-pass prediction with positional priors. Both alternative paradigms produce clearly worse forecasts (FVD-CST above 30, PSNR 20.5–20.6 dB), and MaskGIT in particular loses CST change tracking (Δ-r=0.098).
**Codebook Size:** As a post hoc sensitivity analysis, we replaced the Stage 1 codebook by four smaller FSQ configurations with 240 to 15,360 states (Table 7C). On the test split, all configurations outperform the generative baselines in most metrics, and none dominates the others: two of the smaller codebooks give lower CST change errors (10.6 and 10.7 μm for [8,6,5] and [8,8,8,6,5]) at slightly lower PSNR, while our configuration gives the best L1, PSNR, SSIM and BCVA change error. To separate configuration selection from test evaluation, we repeated the comparison on the validation split with a selection rule fixed in advance (Pareto optimality over PSNR, CST change error and BCVA change error, ties broken by mean rank). On validation, all configurations are statistically indistinguishable (PSNR range 0.19 dB; all CIs of the CST and BCVA differences include zero, except a slightly higher BCVA error for [8,8,8,6,5]); the rule selects [8,5,5,5], which on the test split reaches CST Δ-r=0.81 and a BCVA change error of 3.12 letters, still better than all generative baselines in these metrics (Supplementary Table S6). Performance is therefore robust to the codebook configuration.
**Generator size and reproducibility.** Reducing the Action Transformer to 7.4 M or 1.9 M parameters gives comparable results (CST change error 12.80 and 12.39 vs. 12.72 μm; PSNR 22.45 vs. 22.55 dB), and a 232 M-parameter one-shot generator gives no gain (PSNR 22.57 dB). An independent retraining of the 20.9 M model, evaluated at its final epoch without validation or test inspection, reproduces the reported results (PSNR 22.51 dB, CST change error 12.39 μm, BCVA change error 3.04 letters; all CIs of the differences include zero; Supplementary Table S7). The gains therefore stem from the decoupled latent-action formulation rather than model capacity.

### 3.6. Supplementary Analyses

**Stationary anatomy.** Within the retinal tissue mask, a median of 94.0% of voxels change by less than the inter-visit noise floor (88.1% and 98.7% with *τ* at the 90th and 99th percentiles; Supplementary Table S11, Figure S2). Even among pairs with marked CST change (≥50 μm), 85.6% of tissue voxels are static (78.2–94.2% across thresholds).
**Effect of FSQ on the residual.** Decoding the same encoded residuals of all test pairs with and without FSQ rounding shows that discretization costs negligible fidelity (−0.03 dB PSNR) and maps 28.6% of residual coordinates to exactly zero drift (Supplementary Table S9). The forecast change maps contain less speckle-like high-frequency energy than the observed change (high-frequency share 0.084 vs. 0.103), but this attenuation is essentially identical without rounding (0.085). It therefore arises from the baseline-anchored decoding pathway rather than from FSQ itself.
**Failure cases.** Supplementary Figure S3 shows the top-ranked test pair of pre-defined rules. The observed failure modes are change forecast in stable eyes (e.g., +46 μm forecast vs. +8 μm clinical change over 104 weeks), underestimated fluid resolution (−16 vs. −87 μm over 16 weeks) with loss of the steep baseline retinal tilt in the same case, and image fidelity loss through speckle smoothing and choroidal intensity change despite a correct CST change.

## 4. Discussion

This study presents a two-stage generative framework for forecasting 3D-OCT anatomical change and downstream biomarkers in diabetic retinal disease under anti-VEGF therapy. Our experimental results support three primary observations:

First, our findings clarify the mechanics underlying the failure of standard generative models in longitudinal medical imaging. When monolithic 3D generative networks (such as 3D-adapted CycleGAN, Palette diffusion, and I2I-DiT) are trained directly on full volumes, optimization is dominated by the stationary background anatomy. Treatment-associated fluid changes are local, whereas a median of 94% of retinal tissue voxels change by less than the inter-visit noise floor. Tasked with end-to-end continuous generation, conventional baselines regress to the mean, yielding lower structural similarity (SSIM 0.547–0.567) than a static identity copy (SSIM 0.604). By isolating the longitudinal residual and conditioning the decoder on continuous baseline feature queries, our framework decouples stationary morphology from localized change, achieving the highest structural similarity in every test eye (SSIM 0.657) and a consistent PSNR gain across 52 cross-validated eyes (+1.27 dB over the identity copy).

Second, our experiments demonstrate that standard image fidelity metrics (L1, PSNR, SSIM) do not reliably reflect agreement with downstream biomarkers. While CycleGAN generates visually sharp retinal boundaries, its synthesized volumes decouple from the reference change, yielding a moderate CST change correlation (Δ-r=0.465) and a negative correlation for the OCT-derived BCVA change (Δ-r=−0.166). By contrast, our forecasts track the CST change measured on the real follow-up scans (Δ-r=0.880, Δ-MAE = 12.7 μm on the test split; Δ-r=0.84 across 52 cross-validated eyes), for reference, the 95% limit for a real change between repeated OCT CST measurements is 38 μm [54], and preserve change tracking against the clinical CST labels (Δ-r=0.65, against a regressor ceiling of 0.77). The control experiments show that this depends on patient-specific inputs: replacing the forecast drift by the population prior or by another eye’s drift degrades every metric. For BCVA, the forecasts reproduce the OCT-derived estimate closely (MAE = 3.03 letters), but this estimate itself agrees only moderately with the clinical BCVA change, so BCVA results should be read as a secondary, indirect endpoint.

Third, our analyses clarify the role of Finite Scalar Quantization (FSQ). Learned codebook approaches (VQ-VAE, RQ-VAE) are susceptible to codebook collapse when trained on small clinical cohorts; FSQ provides an explicit, lookup-free rounding scheme that yields a fixed discrete vocabulary at negligible fidelity cost (−0.03 dB) and maps 28.6% of residual coordinates to exactly zero drift. The attenuation of speckle-like high-frequency content in the forecast change arises from the baseline-anchored decoding pathway rather than from FSQ itself. Predicting only the coarse part of each token, anchored by an empirical positional prior, keeps the learned vocabulary small: generators with 1.9–20.9 M parameters perform comparably and a 232 M-parameter generator gives no gain, indicating that the gains stem from the decoupled formulation rather than from model capacity.

**Where the forecasts help and where they fail:** The stratified analyses show that the benefit of our forecasts is concentrated in long follow-up horizons and marked responses, where the identity copy fails (e.g., CST change error 15.4 vs. 29.5 μm beyond 52 weeks). For stable eyes and short intervals, the identity copy is more accurate, because our method tends to forecast some change; the failure cases in Supplementary Figure S3 illustrate this over-prediction, as well as underestimated fluid resolution and the occasional loss of individual retinal contour.
**Clinical Significance and Translational Potential:** In this retrospective study, we demonstrate forecasting of follow-up 3D-OCT volumes and of downstream anatomical biomarkers. Image-based forecasts can be inspected by clinicians, for example, to see where fluid is expected to resolve and whether the foveal contour recovers, which scalar predictions cannot show. Whether such forecasts can support treatment decisions, for example, by flagging eyes at risk of persistent fluid, remains to be established in prospective studies; in particular, the current model is not suited to compare alternative follow-up intervals.
**Limitations and Future Directions:** Several limitations warrant consideration. First, while the OLIVES cohort provides dense longitudinal monitoring under clinical trial protocols, the number of unique eyes is modest (52 time-annotated eyes; five test eyes). We addressed this with eye-level statistics and a 52-eye cross-validation; validation on larger, multi-center cohorts is needed. Second, injection records are available only for PRIME and the injection history is not modeled, so pairs represent follow-up intervals within active anti-VEGF protocols rather than single injection-to-response intervals. Third, our framework forecasts change under fixed anti-VEGF regimens; incorporating multi-agent pharmacotherapy, systemic glycemic biomarkers (e.g., HbA1c), and diabetic duration could further refine individualized predictions. Fourth, CST and BCVA are measured by regressors rather than read from the forecast directly; the BCVA regressor in particular has limited agreement with clinical visual acuity. Fifth, because OLIVES was acquired predominantly on Heidelberg Spectralis devices, both the tokenizer and the downstream regressors may have learned scanner-specific characteristics such as speckle statistics, axial resolution, B-scan spacing and the device’s segmentation conventions for CST. Performance on other devices (e.g., Zeiss Cirrus, Topcon) may therefore differ, and multi-vendor validation, potentially with harmonization or domain adaptation, is required before broader use.

## 5. Conclusions

We presented a two-stage generative forecasting framework for longitudinal 3D-OCT in diabetic retinal disease treated with anti-VEGF therapy. By decoupling baseline ocular anatomy from localized change and quantizing the latent residual via FSQ, the framework avoids the regression-to-the-mean observed in direct 3D generative baselines. Evaluated on the OLIVES clinical trial cohort, our method achieves the highest structural similarity in every test eye (SSIM 0.657, PSNR 22.55 dB) and tracks the CST change measured by a frozen regressor (Δ-r=0.880). In a five-fold cross-validation over 52 eyes, it improves PSNR over an identity copy by 1.27 dB and reduces the CST change error by 10.5 μm, with the largest benefit for long follow-up horizons and marked responses. These results support decoupled latent-action modeling for retrospective OCT forecasting; prospective, multi-center validation is required before any use in treatment decisions.

## Figures and Tables

**Table 1:** Cohort composition of the OLIVES longitudinal dataset and treatment exposure of each regimen. Agents and schedules follow the trial publications [5,46,47]. PRIME excluded eyes with central subfield thickness >320 μm or center-involved DME causing visual loss. DR, diabetic retinopathy; CI-DME, center-involved diabetic macular edema; DRSS, Diabetic Retinopathy Severity Scale; PLI, ultra-widefield panretinal leakage index; PRN, pro re nata.

Regimen	Eyes	Disease at Entry	Agent and Schedule	Time Metadata	Time-Cond.	Train/Val/Test, Eyes (Pairs)
PRIME(NCT03531294)	40	DR (DRSS 47A–71A), no CI-DME	Aflibercept 2.0 mg; monthly until ≥2-step DRSS improvement, then PRN by DRSS or PLI	Exact study week (W0–W104)	Yes	32 (4513)/4 (572)/4 (462)
TREX-DME, Monthly	12	CI-DME	Ranibizumab 0.3 mg every 4 weeks	Nominal, 4-week protocol	Yes	10 (3128)/1 (276)/1 (276)
TREX-DME, TREX	22	CI-DME	Ranibizumab 0.3 mg, treat-and-extend	Visit order; weeks incomplete	No	—
TREX-DME, GILA	22	CI-DME	Ranibizumab 0.3 mg, treat-and-extend + angiography-guided laser	Visit order; weeks incomplete	No	—

**Table 2:** Quantitative comparison on the held-out OLIVES test split (738 pairs from 5 independent eyes). Values are means over pairs; brackets give eye-level 95% confidence intervals from a hierarchical bootstrap (eyes resampled, then pairs within eyes; 2000 replicates). FVD-CST is a distribution-level metric and is reported as a point estimate. CST and BCVA are change metrics computed with the frozen, eye-level cross-validated regressors (Section 2.4); BCVA is an OCT-derived estimate. Bold and underlined values denote the best and second-best point estimates among generative models (* Identity is a non-generative reference). ↑/↓: higher/lower is better. Rows in italics are method categories; the gray background marks our method. Complete metrics for all methods and controls are given in Supplementary Table S1.

Method	Generative Fidelity	Structural Similarity			Downstream Biomarker Evaluation			
	FVD-CST ↓	L1 ↓	PSNR (dB) ↑	SSIM ↑	CST Δ-*r* ↑	CST Δ-MAE (μm) ↓	BCVA Δ-*r* ↑	BCVA Δ-MAE (let.) ↓
*Static Reference*								
Identity (Zero-Op) *	2.5	0.1058	21.98	0.604	—	17.2	—	3.35
		[0.0816, 0.1234]	[19.94, 24.54]	[0.541, 0.697]		[6.6, 27.2]		[1.10, 5.44]
*Adversarial/GAN-Based Solution*								
CycleGAN [37]	**5.0**	0.0989	22.50	0.623	0.465	16.6	−0.166	3.59
		[0.0790, 0.1115]	[20.89, 24.55]	[0.571, 0.705]	[−0.484, 0.671]	[7.7, 25.2]	[−0.286, 0.555]	[1.08, 5.98]
*Diffusion-Based Solutions*								
Palette [39]	21.1	0.1225	20.65	0.547	0.798	14.5	0.739	7.04
		[0.0937, 0.1408]	[19.06, 22.82]	[0.475, 0.656]	[0.213, 0.858]	[9.3, 20.2]	[0.095, 0.793]	[3.06, 11.01]
I2I-DiT [40]	18.0	0.1164	20.71	0.567	0.777	15.0	0.543	3.56
		[0.0972, 0.1349]	[20.01, 21.83]	[0.509, 0.655]	[0.493, 0.821]	[10.2, 18.5]	[0.138, 0.721]	[2.66, 4.21]
*Auto-regressive/Discrete Action Solutions*								
VQGAN [49]	14.8	0.1144	21.12	0.543	0.677	19.3	0.631	4.15
		[0.0881, 0.1318]	[19.63, 23.09]	[0.464, 0.656]	[0.247, 0.735]	[11.1, 24.7]	[0.049, 0.745]	[2.63, 6.27]
**Ours (Decoupled Latent Action)**	15.8	**0.0965**	**22.55**	**0.657**	**0.880**	**12.7**	**0.773**	**3.03**
		[0.0773, 0.1113]	[21.25, 24.53]	[0.607, 0.738]	[0.317, 0.931]	[10.0, 15.4]	[0.169, 0.876]	[2.66, 3.41]

**Table 3:** Paired eye-level comparison with each comparator on the test split (5 eyes). Each cell gives the advantage of our method (Ours−comparator for PSNR and SSIM; comparator−Ours for errors; positive values favor our method), its hierarchical bootstrap 95% CI computed on the same bootstrap replicates for both methods, and the number of eyes in which our method is better. Bold: CI excludes zero. With five eyes, the smallest attainable two-sided exact sign-flip *p* is 0.0625 (all eyes in one direction). The row in italics is a group heading.

Comparator	PSNR (dB)	SSIM	CST Δ-MAE (μm)	BCVA Δ-MAE (let.)
Identity copy	+0.56	**+0.053**	+4.50	+0.32
	[−0.43, 1.37]; 3/5	[0.026, 0.073]; 5/5	[−4.48, 13.97]; 2/5	[−1.70, 2.41]; 1/5
CycleGAN	+0.04	**+0.034**	+3.92	+0.57
	[−0.54, 0.40]; 3/5	[0.019, 0.043]; 5/5	[−5.15, 11.96]; 1/5	[−1.69, 2.97]; 1/5
Palette	**+1.90**	**+0.110**	+1.75	**+4.01**
	[1.30, 2.24]; 5/5	[0.080, 0.137]; 5/5	[−1.06, 5.32]; 4/5	[0.27, 7.96]; 4/5
I2I-DiT	**+1.84**	**+0.090**	+2.30	+0.54
	[1.16, 2.74]; 5/5	[0.081, 0.103]; 5/5	[−0.12, 4.62]; 4/5	[−0.13, 1.19]; 4/5
VQGAN	**+1.43**	**+0.114**	+6.61	+1.13
	[0.82, 1.73]; 5/5	[0.080, 0.148]; 5/5	[−0.60, 11.12]; 4/5	[−0.55, 3.54]; 4/5
*Controls (same decoder and priors; only the drift tokens change)*				
No-conditioning control	**+0.15**	**+0.007**	+4.18	**+0.42**
	[0.03, 0.25]; 5/5	[0.002, 0.011]; 5/5	[−0.03, 6.94]; 3/5	[0.05, 0.73]; 3/5
Shuffled-eye control	**+0.22**	**+0.011**	**+4.43**	**+0.49**
	[0.10, 0.32]; 5/5	[0.007, 0.015]; 5/5	[0.11, 8.39]; 4/5	[0.05, 0.83]; 3/5

**Table 4:** Change-focused evaluation and tests of patient specificity (test split, 738 pairs/5 eyes). The no-conditioning control draws drift tokens from the population positional prior (no image, interval or covariates); the shuffled-eye control uses drift tokens generated for a different eye; both share our decoder and priors. Non-imaging baselines regress the change on baseline CST, BCVA, interval and regimen. Sign: agreement in the direction of change. The last row, in italics, is the regressor applied to the real follow-up scan, i.e., the ceiling of any image-based forecast against the clinical labels.

Method	Image		ΔCST vs. Regressor on Real Scan			ΔCST vs. Clinical Label			ΔBCVA (OCT-Derived)		
	PSNR	SSIM	MAE (μm)	Δ-*r*	Sign	MAE (μm)	Δ-*r*	Sign	MAE (let.)	Δ-*r*	Sign
Identity copy	21.98	0.604	17.2	—	—	12.4	—	0.62	3.35	—	—
No-conditioning control	22.40	0.650	16.9	0.69	0.65	16.7	0.40	0.44	3.44	0.64	0.65
Shuffled-eye control	22.32	0.646	17.1	0.61	0.66	16.6	0.29	0.45	3.51	0.60	0.66
Interval mean (non-imaging)	—	—	19.4	0.29	0.68	19.5	0.22	0.50	3.28	0.31	0.61
Ridge (non-imaging)	—	—	27.8	0.55	0.47	22.9	0.69	0.24	2.68	0.67	0.65
Gradient boosting (non-imaging)	—	—	16.0	0.44	0.68	11.6	0.34	0.64	2.41	0.73	0.68
Ours	22.55	0.657	12.7	0.88	0.69	14.3	0.65	0.53	3.03	0.77	0.66
*Regressor on real follow-up*	—	—	—	—	—	11.3	0.77	0.71	—	—	—

**Table 5:** Five-fold eye-level cross-validation of the forecasting stage over all 52 time-annotated eyes. In each fold, the generator and its empirical priors were re-estimated on the training folds only, with a fixed recipe and no per-fold selection; the Stage 1 tokenizer was kept fixed (subset of eyes unseen by the tokenizer: Supplementary Table S5). Advantage columns: difference (positive favors our method) with hierarchical bootstrap 95% CI, number of eyes in which our method is better, and eye-level sign-flip *p* (Monte Carlo, 20,000 draws). Bold: CI excludes zero. ↑/↓: higher/lower is better. The row in italics is a group heading.

Metric	Ours	Identity	Advantage vs. Identity	No-Cond. Control	Advantage vs. Control
*All 52 eyes (9227 pairs)*					
PSNR (dB) ↑	24.07	22.79	**+1.27**	23.94	**+0.13**
			[0.93, 1.57]; 46/52, p<0.001		[0.03, 0.22]; 34/52, p=0.004
L1 ↓	0.0867	0.1002	**+0.0135**	0.0879	**+0.0012**
			[0.0093, 0.0173]; 45/52, p<0.001		[0.0000, 0.0024]; 35/52, p=0.026
CST Δ-MAE, regressor (μm) ↓	17.93	28.41	**+10.48**	19.54	**+1.61**
			[3.07, 19.37]; 31/52, p=0.009		[0.04, 3.44]; 29/52, p=0.053
CST Δ-MAE, clinical label (μm) ↓	19.58	28.15	**+8.57**	21.21	+1.63
			[0.30, 18.70]; 22/52, p=0.202		[−0.16, 3.81]; 27/52, p=0.128
BCVA Δ-MAE, OCT-derived (let.) ↓	2.19	2.53	+0.34	2.23	+0.05
			[−0.03, 0.69]; 32/52, p=0.688		[−0.10, 0.16]; 35/52, p=0.758
CST Δ-*r*, regressor ↑	0.841	—	—	0.793	**+0.048**
	[0.693, 0.895]			[0.646, 0.860]	[0.022, 0.081]

**Table 6:** Performance by follow-up horizon and by magnitude of the clinically measured CST change (test split). Cells: CST Δ-MAE (μm, frozen regressor)/Δ-*r*; column headers give pairs/eyes. Bold: Lowest error in each stratum. Strata by magnitude use the clinical label; <10 μm is close to the test–retest difference of OCT CST measurements. Label-space results: Supplementary Table S10.

Method	Follow-Up Horizon				Magnitude of Clinical |ΔCST|		
	≤12 w (198/5)	13–26 w (163/5)	27–52 w (235/5)	>52 w (142/3)	<10 μm (458/5)	10–25 μm (185/5)	≥25 μm (95/3)
**Ours**	10.1/0.60	**11.4**/0.77	**14.2**/0.88	**15.4** / 0.96	10.2/0.72	**13.3**/0.87	23.9/0.93
Identity copy	8.5/—	12.9/—	20.1/—	29.5/—	9.1/—	21.7/—	47.3/—
Palette	13.0/0.58	12.8/0.73	15.1/0.83	17.4/0.91	11.7/0.52	17.3/0.73	22.2/0.88
I2I-DiT	12.4/0.53	12.3/0.72	17.6/0.72	17.5/0.88	12.8/0.54	17.8/0.56	**20.4**/0.92
CycleGAN	**8.3**/0.34	13.0/0.12	19.8/0.30	27.1/0.67	9.2/0.34	21.6/0.09	42.7/0.76
VQGAN	13.0/0.43	15.1/0.63	21.6/0.71	29.3/0.79	14.0/0.48	22.3/0.66	39.2/0.86
No-conditioning control	11.9/0.55	13.3/0.73	18.5/0.76	25.4/0.76	12.4 /0.71	15.7/0.83	41.0/0.71
Shuffled-eye control	11.0/0.56	13.8/0.64	19.1/0.67	26.2/0.70	11.6/0.70	17.1/0.79	44.0/0.49
Gradient boosting (non-imaging)	8.6/0.19	12.1/0.24	18.5/0.43	26.5/0.84	**8.4**/0.41	20.1/0.42	44.3/0.40

**Table 7:** Ablation analyses on the held-out test split (738 pairs/5 eyes). All analyses were run after the main model had been fixed and were not used to select it. Group B compares complete Stage 2 generation strategies (trainable Stage 2 parameters in parentheses). Group C varies the Stage 1 codebook; on the validation split all configurations are statistically indistinguishable (Supplementary Table S6). Bold: Best within each group. ↑/↓: higher/lower is better. Rows in italics are group headings.

Design Configuration	Gen. Fidelity	Structural Similarity			Downstream Biomarker Evaluation			
	FVD-CST↓	L1↓	PSNR↑	SSIM↑	CST Δ-*r* ↑	CST Δ-MAE↓	BCVA Δ-*r* ↑	BCVA Δ-MAE ↓
***A. Target formulation***								
Direct Pixel Synthesis	**14.3**	0.1128	21.24	0.556	0.671	16.3	**0.777**	3.17
**Latent residual (Ours)**	15.8	**0.0965**	**22.55**	**0.657**	**0.880**	**12.7**	0.773	**3.03**
***B. Stage 2 generation paradigm** (shared tokenizer and baseline-query decoder)*								
Autoregressive decoding (363.4 M)	30.9	0.1345	20.60	0.651	0.665	16.4	0.703	4.10
MaskGIT iterative unmasking (363.4 M)	31.3	0.1347	20.51	0.648	0.098	17.1	0.768	3.31
**Single pass + positional prior (Ours, 20.9 M)**	**15.8**	**0.0965**	**22.55**	**0.657**	**0.880**	**12.7**	**0.773**	**3.03**
***C. FSQ codebook size** (post hoc sensitivity analysis; validation results in Supplementary Table S6)*								
[8, 6, 5] (240 states)	**15.5**	0.0988	22.47	0.648	0.894	**10.6**	0.771	3.57
[8, 5, 5, 5] (1000 states)	16.9	0.0979	22.47	0.648	0.810	14.6	0.672	3.12
[7, 5, 5, 5, 5] (4375 states)	17.3	0.0972	22.43	0.649	0.838	14.5	0.697	3.68
[8, 8, 8, 6, 5] (15,360 states)	16.3	0.0987	22.42	0.652	**0.915**	10.7	**0.813**	3.30
[8, 8, 8, 5, 5, 5] **(64,000 states; Ours, fixed a priori)**	15.8	**0.0965**	**22.55**	**0.657**	0.880	12.7	0.773	**3.03**

## Data Availability

The OLIVES dataset analyzed in this study is publicly accessible via the official GitHub repository at https://github.com/olivesgatech/OLIVES_Dataset (accessed on 13 October 2026), Zenodo (https://doi.org/10.5281/zenodo.7105232 (accessed on 13 October 2026)), and HuggingFace (https://huggingface.co/datasets/gOLIVES/OLIVES_Dataset (accessed on 13 October 2026)). Complete source code and trained model checkpoints will be made publicly available on GitHub upon formal publication.

## Supplementary Materials

The following supporting information can be downloaded at: https://www.mdpi.com/article/10.3390/diagnostics16203382/s1: Figure S1: From latent residual to action tokens and their prediction; Figure S2: Quantification of stationary anatomy; Figure S3: Rule-based success and failure cases (central B-scan); Table S1: Complete test-split metrics (738 pairs/5 eyes) with eye-level hierarchical-bootstrap 95% CIs for all methods and controls; Table S2: Downstream regressors used for CST and OCT-derived BCVA evaluation; Table S3: Sensitivity to the pairing strategy (test split, 5 eyes); Table S4: Evaluation against the clinical CST and BCVA labels recorded in OLIVES (test split, 738 pairs/5 eyes); Table S5: Cross-validation results for all methods, including non-imaging baselines fitted per fold on the training eyes; Table S6: FSQ codebook analysis on the validation split with a selection rule fixed in advance; Table S7: Generator-size ablation and independent retraining; Table S8: Implementation details and parameter counts of the final model; Table S9: Effect of FSQ on the latent residual (test split); Table S10: Stratified results against the clinical CST labels (test split); Table S11: Fraction of voxels that change by less than the inter-visit noise floor τ (median/10th percentile over pairs).
