# Supplementary material for: Generative Forecasting of Longitudinal 3D-OCT Under Anti-VEGF Therapy for Diabetic Macular Edema
Source: Diagnostics (Basel). 2026 Oct 17;16(20):3382. doi: 10.3390/diagnostics16203382 (PMC13703180; doi:10.3390/diagnostics16203382)
Supplement: Supplementary file 1 [file diagnostics-16-03382-s001.zip › diagnostics-4597450-supplementary.pdf]

Supplementary Materials

Generative Forecasting of Longitudinal 3D-OCT Under Anti-VEGF Therapy for Diabetic Macular Edema

Yuxiang Feng, Chaofeng Liang and Shujun Wang

Supplementary Figures

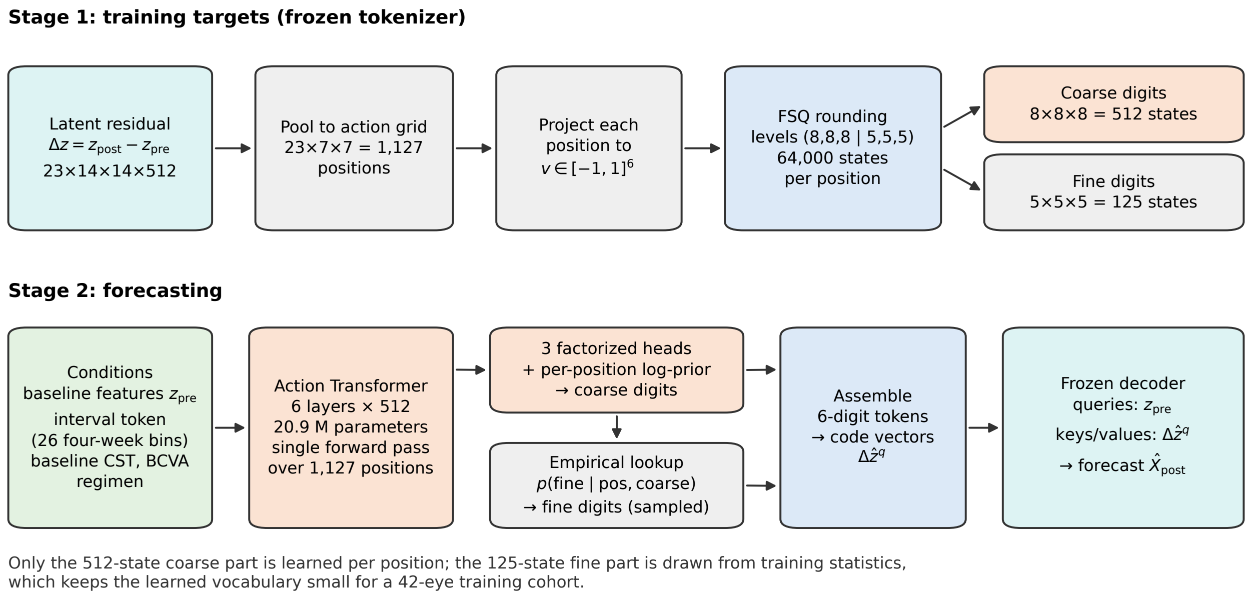

Figure S1: From latent residual to action tokens and their prediction. Each of the 1,127 action positions carries one FSQ token from 64,000 implicit states; the Action Transformer predicts the 512-state coarse part with three factorized heads anchored by a per-position prior, and the 125-state fine part is sampled from an empirical conditional table estimated on training pairs.

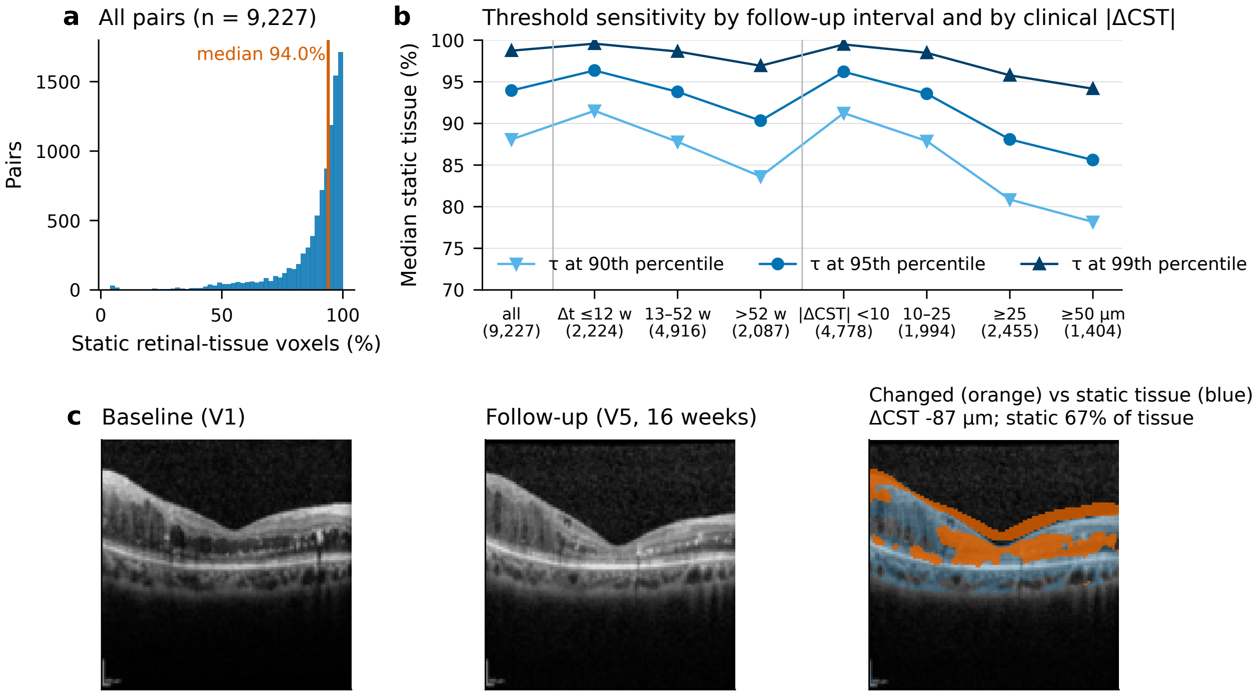

Figure S2: Quantification of stationary anatomy. (a) Distribution of the fraction of retinal-tissue voxels whose smoothed change stays below the inter-visit noise floor τ (95th percentile of change in 260 unchanged reference pairs). (b) Median static fraction by interval and by clinical $|\Delta\text{CST}|$ for three choices of τ. (c) Example test pair with a marked CST decrease: even here most tissue voxels are static, and change is concentrated around the fovea and inner retina.

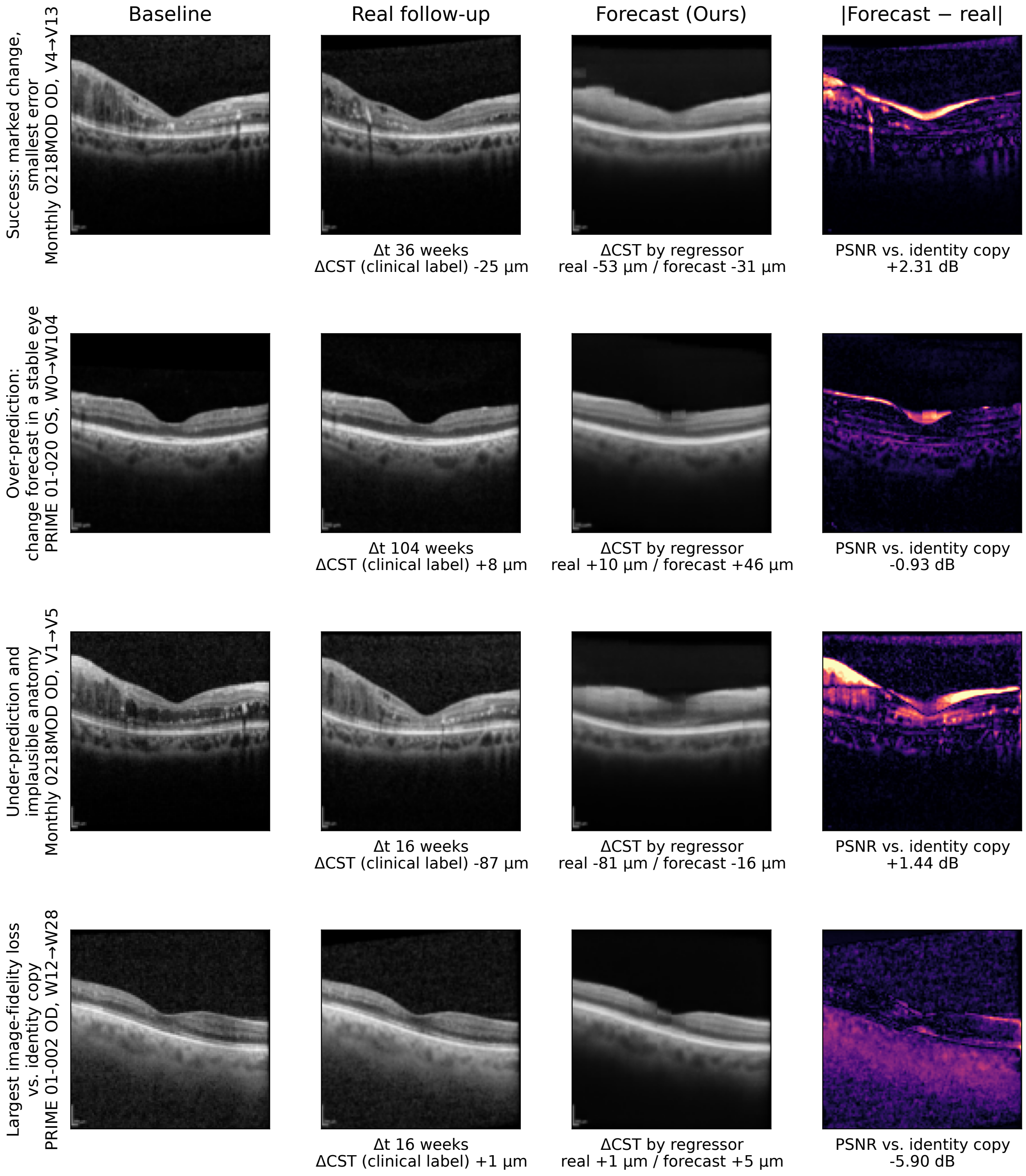

Figure S3: Rule-based success and failure cases (central B-scan). Each row is the top-ranked test pair of a pre-defined rule, with at most one case per eye per category: success, $|\Delta\text{CST}| \geq 25\,\mu$m with the smallest error; over-prediction, $|\Delta\text{CST}| < 10\,\mu$m with the largest forecast change; under-prediction, $\Delta\text{CST} \leq -25\,\mu$m with the largest underestimate; image fidelity, the largest PSNR loss relative to the identity copy. Rows 2–4 show change forecast in a stable eye, underestimated fluid resolution with loss of the baseline retinal tilt (regression toward an average contour), and image-fidelity loss through speckle smoothing and choroidal intensity change although the CST change is correct. Each row reports the clinical ΔCST label and the regressor's ΔCST on the real and the forecast scan, so that errors of the forecast and of the regressor can be told apart.

Supplementary Tables

Table S1: Complete test-split metrics (738 pairs / 5 eyes) with eye-level hierarchical-bootstrap 95% CIs for all methods and controls. CST/BCVA: frozen cross-validated regressors.

Method	L1	PSNR	SSIM	CST Δ-MAE	CST Δ-r	CST sign	BCVA Δ-MAE	BCVA Δ-r	BCVA sign
Identity copy	0.1058 [0.0816, 0.1234]	21.98 [19.94, 24.54]	0.604 [0.541, 0.697]	17.22 [6.58, 27.23]	—	—	3.35 [1.10, 5.44]	—	—
CycleGAN	0.0989 [0.0790, 0.1115]	22.50 [20.89, 24.55]	0.623 [0.571, 0.705]	16.64 [7.68, 25.21]	0.465 [−0.484, 0.671]	0.51 [0.40, 0.65]	3.59 [1.08, 5.98]	−0.166 [−0.286, 0.555]	0.49 [0.39, 0.62]
Palette	0.1225 [0.0937, 0.1408]	20.65 [19.06, 22.82]	0.547 [0.475, 0.656]	14.48 [9.28, 20.25]	0.798 [0.213, 0.858]	0.63 [0.43, 0.78]	7.04 [3.06, 11.01]	0.739 [0.095, 0.793]	0.66 [0.46, 0.81]
I2I-DiT	0.1164 [0.0972, 0.1349]	20.71 [20.01, 21.83]	0.567 [0.509, 0.655]	15.03 [10.22, 18.51]	0.777 [0.493, 0.821]	0.66 [0.50, 0.77]	3.56 [2.66, 4.21]	0.543 [0.138, 0.721]	0.62 [0.46, 0.74]
VQGAN	0.1144 [0.0881, 0.1318]	21.12 [19.63, 23.09]	0.543 [0.464, 0.656]	19.33 [11.14, 24.70]	0.677 [0.247, 0.735]	0.59 [0.43, 0.68]	4.15 [2.63, 6.27]	0.631 [0.049, 0.745]	0.70 [0.56, 0.82]
No-conditioning control	0.0983 [0.0781, 0.1130]	22.40 [21.01, 24.43]	0.650 [0.599, 0.733]	16.90 [12.48, 20.28]	0.691 [0.384, 0.744]	0.65 [0.46, 0.78]	3.44 [3.12, 3.77]	0.636 [0.155, 0.764]	0.65 [0.45, 0.82]
Shuffled-eye control	0.0993 [0.0791, 0.1139]	22.32 [20.95, 24.34]	0.646 [0.595, 0.728]	17.15 [11.47, 21.84]	0.610 [0.399, 0.692]	0.66 [0.45, 0.79]	3.51 [3.20, 3.82]	0.596 [0.156, 0.730]	0.66 [0.47, 0.82]
Ours	0.0965 [0.0773, 0.1113]	22.55 [21.25, 24.53]	0.657 [0.607, 0.738]	12.72 [9.97, 15.42]	0.880 [0.317, 0.931]	0.69 [0.47, 0.84]	3.03 [2.66, 3.41]	0.773 [0.169, 0.876]	0.66 [0.46, 0.82]

Table S2: Downstream regressors used for CST and OCT-derived BCVA evaluation.

Target	Volumes / eyes	Initialization	Fine-tuned	LR backbone / head	Epochs (patience)	Pooled OOF r [fold range]	Pooled OOF MAE
CST	670 / 73	RETFound-OCT (MAE)	last 24 blocks	1e-5 / 1e-3	60 (12)	0.958 [0.94–0.97]	17.8 μm
BCVA	706 / 73	RETFound-OCT (MAE)	last 24 blocks	1e-5 / 1e-3	20 (5)	0.577 [0.35–0.74]	7.0 letters

Training used 5-fold eye-level cross-validation (seed 42). At evaluation every volume (baseline, real follow-up, forecast) is scored by the fold model whose training data did not contain that eye; eyes outside the 73 labelled eyes are scored by the mean of the five fold models. Hyper-parameters were chosen on pooled out-of-fold agreement with the clinical labels only, without reference to any generative model; all methods are scored with the same final regressors. Change-tracking ceiling (regressor on real follow-up vs. clinical labels, Δ-r): CST 0.77 (test, 5 eyes) / 0.93 (52-eye CV); BCVA 0.40 / 0.26.

Table S3: Sensitivity to the pairing strategy (test split, 5 eyes). Consecutive: adjacent visits only (no intermediate visits). Baseline-anchored: first visit → each later visit. Cells: CST Δ-MAE (μm) / Δ-r.

Method	vs. regressor on real scan			vs. clinical label		
	All (738)	Consecutive (80)	Baseline-anchored (80)	All (738)	Consecutive (80)	Baseline-anchored (80)
Ours	12.7 / 0.88	9.8 / 0.61	30.4 / 0.97	14.3 / 0.65	11.7 / 0.28	28.5 / 0.95
Identity copy	17.2 / —	8.3 / —	42.3 / —	12.4 / —	6.5 / —	39.5 / —
Palette	14.5 / 0.80	12.7 / 0.56	20.6 / 0.91	14.8 / 0.65	14.2 / 0.34	18.2 / 0.91
I2I-DiT	15.0 / 0.78	11.8 / 0.54	19.8 / 0.96	15.5 / 0.65	13.4 / 0.24	18.5 / 0.93
CycleGAN	16.6 / 0.46	8.0 / 0.45	34.4 / 0.85	13.2 / 0.33	7.7 / −0.02	31.5 / 0.80
VQGAN	19.3 / 0.68	13.0 / 0.47	34.3 / 0.88	17.4 / 0.47	12.9 / 0.29	32.1 / 0.85
No-conditioning control	16.9 / 0.69	12.0 / 0.45	42.5 / 0.84	16.7 / 0.40	13.6 / 0.20	40.6 / 0.85
Shuffled-eye control	17.1 / 0.61	11.8 / 0.44	45.4 / 0.73	16.6 / 0.29	13.5 / 0.19	43.4 / 0.70
Gradient boosting (non-imaging)	16.0 / 0.44	8.7 / 0.15	39.7 / 0.18	11.6 / 0.34	6.8 / 0.19	36.2 / 0.19

Table S4: Evaluation against the clinical CST and BCVA labels recorded in OLIVES (test split, 738 pairs / 5 eyes). Forecast change = regressor(forecast) − regressor(baseline); true change = label difference. The last row applies the same regressor to the real follow-up scan and bounds what any image-based forecast can reach.

Method	ΔCST vs. clinical label (μm)				ΔBCVA vs. clinical label (letters)			
	MAE	Δ-r	Sign	Advantage of Ours	MAE	Δ-r	Sign	Advantage of Ours
Identity copy	12.40 [5.05, 17.85]	—	0.62	−1.89 [−9.30, 3.19]; 2/5	4.13 [3.12, 4.86]	—	0.64	−0.88 [−1.86, −0.11]; 0/5
CycleGAN	13.17 [7.72, 17.42]	0.334 [−0.413, 0.578]	0.64	−1.12 [−6.89, 2.56]; 1/5	4.62 [3.39, 5.58]	−0.257 [−0.343, 0.020]	0.64	−0.39 [−1.41, 0.46]; 1/5
Palette	14.83 [9.29, 23.79]	0.649 [−0.065, 0.828]	0.52	+0.54 [−1.86, 3.90]; 4/5	8.67 [4.42, 12.63]	0.317 [−0.249, 0.593]	0.59	+3.66 [0.09, 7.49]; 4/5
I2I-DiT	15.54 [11.20, 19.96]	0.652 [0.184, 0.762]	0.44	+1.25 [−1.18, 2.95]; 3/5	4.82 [4.07, 5.50]	0.153 [−0.219, 0.327]	0.65	−0.19 [−0.53, 0.36]; 3/5
VQGAN	17.42 [11.90, 25.49]	0.469 [−0.025, 0.637]	0.47	+3.13 [−1.72, 7.38]; 4/5	5.52 [3.57, 8.34]	0.229 [−0.154, 0.525]	0.52	+0.51 [−1.13, 2.98]; 3/5
No-conditioning control	16.75 [14.13, 20.02]	0.400 [0.074, 0.517]	0.44	+2.46 [−0.21, 4.09]; 3/5	4.88 [3.90, 5.72]	0.171 [−0.254, 0.472]	0.61	−0.13 [−0.44, 0.18]; 2/5
Shuffled-eye control	16.58 [13.20, 20.36]	0.295 [0.075, 0.426]	0.45	+2.29 [0.06, 3.98]; 4/5	4.81 [3.82, 5.81]	0.153 [−0.228, 0.440]	0.61	−0.20 [−0.66, 0.30]; 2/5
Ridge (non-imaging)	22.92 [12.82, 28.94]	0.689 [0.381, 0.847]	0.24	+8.63 [−3.99, 15.51]; 3/5	3.64 [3.31, 4.04]	0.439 [−0.015, 0.735]	0.64	−1.37 [−1.90, −0.47]; 1/5
Gradient boosting (non-imaging)	11.62 [4.26, 17.14]	0.343 [0.168, 0.733]	0.64	−2.67 [−9.83, 1.85]; 1/5	4.27 [3.50, 4.86]	0.355 [−0.142, 0.781]	0.67	−0.75 [−1.45, −0.17]; 0/5
Ours	14.29 [10.86, 20.09]	0.649 [0.062, 0.803]	0.53	—	5.01 [3.97, 5.68]	0.259 [−0.336, 0.586]	0.64	—
Regressor on real follow-up	11.27 [4.74, 16.11]	0.767 [0.297, 0.867]	0.71	−3.02 [−9.31, 2.89]; 1/5	4.00 [2.94, 4.62]	0.401 [−0.077, 0.597]	0.71	−1.02 [−1.50, −0.60]; 1/5

Table S5: Cross-validation results for all methods, including non-imaging baselines fitted per fold on the training eyes.

Method	CST Δ-MAE, regressor		CST Δ-MAE, label		BCVA Δ-MAE	
	Value	Advantage of Ours	Value	Advantage of Ours	Value	Advantage of Ours
All 52 eyes						
Ours	17.93 [13.54, 22.75]	—	19.58 [14.70, 24.51]	—	2.19 [1.85, 2.55]	—
Identity copy	28.41 [18.74, 39.76]	+10.48 [3.07, 19.37]; 31/52, $p = 0.009$	28.15 [16.72, 41.51]	+8.57 [0.30, 18.70]; 22/52, $p = 0.202$	2.53 [1.99, 3.12]	+0.34 [−0.03, 0.69]; 32/52, $p = 0.688$
No-conditioning control	19.54 [14.49, 24.96]	+1.61 [0.04, 3.44]; 29/52, $p = 0.053$	21.21 [15.50, 27.27]	+1.63 [−0.16, 3.81]; 27/52, $p = 0.128$	2.23 [1.91, 2.58]	+0.05 [−0.10, 0.16]; 35/52, $p = 0.758$
Interval mean (non-imaging)	32.63 [25.43, 41.53]	+14.70 [9.27, 21.32]; 41/52, $p < 0.001$	32.43 [23.87, 43.26]	+12.85 [7.04, 20.71]; 38/52, $p < 0.001$	2.49 [2.05, 2.99]	+0.30 [0.03, 0.56]; 34/52, $p = 0.468$
Ridge (non-imaging)	24.68 [19.25, 30.21]	+6.75 [3.70, 10.26]; 42/52, $p < 0.001$	23.70 [17.86, 29.58]	+4.12 [1.06, 7.31]; 36/52, $p = 0.002$	2.49 [2.08, 2.94]	+0.30 [0.06, 0.52]; 33/52, $p = 0.104$
Gradient boosting (non-imaging)	20.34 [15.17, 25.79]	+2.41 [−0.47, 5.33]; 29/52, $p = 0.245$	19.06 [12.98, 26.05]	−0.52 [−3.63, 3.14]; 17/52, $p = 0.182$	2.82 [2.33, 3.42]	+0.63 [0.27, 1.00]; 35/52, $p = 0.009$
Regressor on real follow-up	—	—	11.17 [8.51, 14.09]	−8.41 [−12.56, −4.50]; 6/52, $p < 0.001$	—	—
Tokenizer-unseen eyes (10)						
Ours	16.66 [10.72, 21.85]	—	18.74 [11.47, 25.56]	—	2.44 [1.82, 2.98]	—
Identity copy	20.92 [8.83, 31.52]	+4.26 [−3.20, 11.65]; 4/10, $p = 0.604$	17.44 [7.59, 26.47]	−1.30 [−5.67, 2.73]; 3/10, $p = 0.188$	2.78 [1.37, 4.31]	+0.34 [−0.86, 1.55]; 4/10, $p = 0.664$
No-conditioning control	17.41 [11.65, 22.19]	+0.75 [−1.11, 2.40]; 5/10, $p = 0.529$	18.46 [12.50, 23.96]	−0.28 [−2.29, 1.82]; 4/10, $p = 0.811$	2.61 [1.91, 3.24]	+0.17 [−0.02, 0.37]; 8/10, $p = 0.090$
Interval mean (non-imaging)	23.94 [16.51, 31.49]	+7.28 [1.79, 12.79]; 5/10, $p = 0.064$	23.26 [16.29, 31.30]	+4.52 [−2.43, 11.01]; 5/10, $p = 0.557$	2.81 [1.86, 3.84]	+0.38 [−0.46, 1.14]; 5/10, $p = 0.826$
Ridge (non-imaging)	27.78 [16.11, 41.82]	+11.11 [1.55, 24.76]; 8/10, $p = 0.031$	25.55 [15.67, 35.45]	+6.80 [−2.89, 18.79]; 8/10, $p = 0.307$	2.34 [1.58, 3.09]	−0.09 [−0.73, 0.39]; 4/10, $p = 0.221$
Gradient boosting (non-imaging)	16.22 [8.82, 22.65]	−0.44 [−3.79, 3.75]; 3/10, $p = 0.363$	13.84 [7.36, 20.22]	−4.90 [−9.11, −1.06]; 1/10, $p = 0.010$	2.36 [1.62, 3.11]	−0.08 [−0.64, 0.42]; 5/10, $p = 0.471$
Regressor on real follow-up	—	—	9.86 [5.93, 13.37]	−8.88 [−15.07, −1.90]; 1/10, $p = 0.006$	—	—

Table S6: FSQ codebook analysis on the validation split with a selection rule fixed in advance. Objectives: PSNR ↑, CST Δ-MAE ↓, BCVA Δ-MAE ↓; a configuration is Pareto-optimal if no other is at least as good on all three and strictly better on one; ties are broken by the lowest mean rank (†: rule-selected). "vs. Ours" gives the configuration's error minus ours with hierarchical-bootstrap 95% CI (positive: ours better; bold: CI excludes zero). All configurations are statistically indistinguishable on validation (PSNR range 0.19 dB), except a slightly higher BCVA error for [8,8,8,6,5].

Levels	Codebook	Validation (848 pairs / 5 eyes)							Test		
		PSNR	CST Δ-MAE	vs. Ours	BCVA Δ-MAE	vs. Ours	Pareto	Mean rank	PSNR	CST Δ-MAE / Δ-r	BCVA Δ-MAE / Δ-r
[8,6,5]	240	23.34	18.53	+0.75 [−2.27, 5.06]; 3/5	1.89	−0.08 [−0.42, 0.22]; 2/5	✓	3.67	22.47	10.55 / 0.894	3.57 / 0.771
[8,5,5,5]†	1,000	23.53	17.40	−0.38 [−1.55, 0.89]; 2/5	1.93	−0.04 [−0.28, 0.19]; 2/5	✓	1.67	22.47	14.58 / 0.810	3.12 / 0.672
[7,5,5,5,5]	4,375	23.42	16.99	−0.79 [−1.71, 0.29]; 1/5	2.00	+0.03 [−0.11, 0.18]; 4/5	✓	2.67	22.43	14.48 / 0.838	3.68 / 0.697
[8,8,8,6,5]	15,360	23.36	17.57	−0.21 [−1.85, 1.51]; 4/5	2.33	+0.35 [0.08, 0.64]; 4/5		4.00	22.42	10.70 / 0.915	3.30 / 0.813
[8,8,8,5,5,5] (Ours)	64,000	23.46	17.77	—	1.97	—		3.00	22.55	12.72 / 0.880	3.03 / 0.773

Table S7: Generator-size ablation and independent retraining. All variants use the same recipe (10 epochs, final-epoch checkpoint, no validation or test inspection). CST diff.: variant Δ-MAE minus that of the reported model; all CIs include zero. A larger generator is listed for reference (‡evaluated with an earlier sampling harness; image metrics only).

Generator	Params (M)	Validation			Test				
		PSNR	CST Δ-MAE / Δ-r	BCVA Δ-MAE	PSNR	SSIM	CST Δ-MAE / Δ-r	CST diff. vs. reported [95% CI]	BCVA Δ-MAE / Δ-r
2 layers × 256	1.88	23.45	18.14 / 0.793	1.91	22.45	0.656	12.39 / 0.859	−0.33 [−0.87, 0.32]	3.54 / 0.708
4 layers × 384	7.37	23.49	17.26 / 0.809	1.94	22.45	0.655	12.80 / 0.854	+0.08 [−1.13, 1.05]	3.03 / 0.762
6 layers × 512, independent retraining (final epoch)	20.87	23.45	17.56 / 0.808	2.01	22.51	0.658	12.39 / 0.869	−0.33 [−1.33, 0.45]	3.04 / 0.752
6 layers × 512 (reported model)	20.87	23.46	17.77 / 0.808	1.97	22.55	0.657	12.72 / 0.880	—	3.03 / 0.773
Full-digit one-shot generator (18 layers × 1024)‡	232.4	—	—	—	22.57	—	—	—	—

Table S8: Implementation details and parameter counts of the final model.

Item	Setting
Stage 1: Retinal Response Tokenizer (frozen in Stage 2)	
Input volume	$46 \times 112 \times 112$, intensities scaled to $[-1, 1]$
Patch size / token grid	$(2, 8, 8)$ / $23 \times 14 \times 14 \times 512$
Encoder / decoder	factorized spatial–depth transformer, 8 + 8 layers, 8 heads, dim 512
Action grid	$23 \times 7 \times 7 = 1{,}127$ positions, 6-d FSQ code per position
FSQ levels	$(8, 8, 8, 5, 5, 5)$, 64,000 implicit states
Stage 2: Action Transformer	
Architecture	6 layers, 8 heads, hidden size 512; single forward pass over 1,127 positions
Prediction targets	3 coarse digits $(8, 8, 8)$, 3 factorized heads + per-position log-prior
Fine digits	sampled from empirical $p(\text{fine} \mid \text{position}, \text{coarse})$ (training pairs)
Conditions	baseline features, interval token (26 four-week bins), baseline CST, BCVA, regimen
Trainable parameters	20.9 M
Optimizer	AdamW, $\beta = (0.9, 0.95)$, learning rate 10^{-4}, weight decay 0.05
Regularization	dropout 0.2; paired horizontal flip ($p = 0.5$)
Batch / epochs	global batch 96 (6 GPUs × 16), 10 epochs, bf16 mixed precision
Training time	≈3 min on 6 × NVIDIA L20X
Checkpoint	retained by the validation-loss callback (top-3)
Sampling	one sample per pair; fp32 decoding through the frozen Stage-1 decoder

Table S9: Effect of FSQ on the latent residual (test split). The same encoded residual of each pair is decoded with and without FSQ rounding through the frozen decoder. High-frequency share: fraction of change-map energy ($X - X_{\text{pre}}$) in the 2D Laplacian band of each B-scan. FSQ zero drift: fraction of residual coordinates rounded to the centre level.

Subset	Pairs	PSNR vs. real follow-up (dB)			High-frequency share of change map			FSQ zero drift
		Identity	Continuous Δz	FSQ Δz	Observed	Continuous	FSQ	
All test pairs	738	21.98	23.93	23.90	0.103	0.085	0.084	28.6%
Stable ($\lvert\Delta\text{CST}\rvert < 5\,\mu$m)	338	22.86	24.58	24.55	0.108	0.093	0.092	29.4%
Changed ($\lvert\Delta\text{CST}\rvert \geq 25\,\mu$m)	95	19.09	22.06	22.04	0.075	0.053	0.052	25.7%

Table S10: Stratified results against the clinical CST labels (test split). Cells: CST Δ-MAE (μm) / Δ-r. Bold: lowest error in each stratum (excluding the regressor ceiling).

Method	Follow-up horizon				Magnitude of clinical $\lvert\Delta\text{CST}\rvert$		
	≤12 w (198/5)	13–26 w (163/5)	27–52 w (235/5)	>52 w (142/3)	<10 μm (458/5)	10–25 μm (185/5)	≥25 μm (95/3)
Ours	12.3 / 0.23	14.4 / 0.41	15.4 / 0.63	15.0 / 0.82	10.5 / 0.27	16.1 / 0.34	29.0 / 0.76
Identity copy	6.7 / —	10.8 / —	13.7 / —	19.9 / —	3.2 / —	15.1 / —	51.7 / —
Palette	14.6 / 0.35	15.6 / 0.54	15.3 / 0.65	13.6 / 0.81	10.8 / 0.21	19.8 / 0.22	24.5 / 0.79
I2I-DiT	13.6 / 0.28	15.2 / 0.48	16.9 / 0.61	16.4 / 0.81	12.6 / 0.22	17.7 / 0.29	25.2 / 0.76
CycleGAN	8.2 / −0.08	12.8 / −0.08	14.7 / 0.23	18.1 / 0.65	5.5 / −0.04	14.8 / −0.16	46.8 / 0.61
VQGAN	13.2 / 0.20	16.3 / 0.27	18.4 / 0.44	23.0 / 0.66	11.5 / 0.19	19.8 / 0.32	41.6 / 0.69
No-conditioning control	13.7 / 0.24	15.9 / 0.36	17.0 / 0.40	21.5 / 0.50	12.0 / 0.32	14.0 / 0.44	44.9 / 0.56
Shuffled-eye control	13.3 / 0.23	15.9 / 0.23	17.3 / 0.27	20.8 / 0.42	11.1 / 0.26	14.3 / 0.41	47.5 / 0.34
Gradient boosting (non-imaging)	6.9 / 0.20	10.2 / 0.26	12.9 / 0.31	17.8 / 0.60	3.8 / 0.25	12.5 / 0.37	47.7 / 0.38
Regressor on real follow-up	8.5 / 0.41	10.7 / 0.59	12.3 / 0.77	14.1 / 0.87	8.4 / 0.34	14.9 / 0.58	18.0 / 0.82

Table S11: Fraction of voxels that change by less than the inter-visit noise floor τ (median / 10th percentile over pairs). τ is the 95th percentile of smoothed ($\sigma = 1$ voxel) voxel-wise change within retinal tissue in 260 anatomically unchanged reference pairs (interval ≤4 weeks, $\lvert\Delta\text{CST}\rvert \leq 2\,\mu$m); tissue = Otsu mask. Last column: tissue-only static fraction with τ at the 90th / 95th / 99th percentile.

Subset	Pairs (eyes)	Whole volume	Retinal tissue	Tissue, τ at q90 / q95 / q99
All time-annotated pairs	9227 (52)	98.0% / 89.7%	94.0% / 71.5%	88.1% / 94.0% / 98.7%
Test split	738 (5)	97.3% / 89.0%	92.0% / 65.9%	84.9% / 92.0% / 97.9%
Interval ≤12 weeks	2224 (52)	98.8% / 93.4%	96.4% / 80.9%	91.5% / 96.4% / 99.6%
Interval 13–52 weeks	4916 (51)	98.0% / 89.9%	93.8% / 71.7%	87.8% / 93.8% / 98.6%
Interval >52 weeks	2087 (41)	96.9% / 86.5%	90.3% / 61.3%	83.6% / 90.3% / 96.9%
$\lvert\Delta\text{CST}\rvert < 10\,\mu$m	4778 (52)	98.8% / 93.4%	96.2% / 81.6%	91.2% / 96.2% / 99.5%
$\lvert\Delta\text{CST}\rvert$ 10–25 μm	1994 (50)	97.9% / 90.8%	93.6% / 74.7%	87.9% / 93.6% / 98.5%
$\lvert\Delta\text{CST}\rvert \geq 25\,\mu$m	2455 (32)	95.9% / 84.1%	88.1% / 53.2%	80.9% / 88.1% / 95.8%
$\lvert\Delta\text{CST}\rvert \geq 50\,\mu$m	1404 (18)	95.2% / 82.2%	85.6% / 47.9%	78.2% / 85.6% / 94.2%
